# Cellular Clearance and Biological Activity of Calciprotein Particles Depend on Their Maturation State and Crystallinity

**DOI:** 10.3389/fimmu.2018.01991

**Published:** 2018-09-04

**Authors:** Sina Köppert, Andrea Büscher, Anne Babler, Ahmed Ghallab, Eva M. Buhl, Eicke Latz, Jan G. Hengstler, Edward R. Smith, Willi Jahnen-Dechent

**Affiliations:** ^1^Helmholtz-Institute for Biomedical Engineering, RWTH Aachen University Hospital, Aachen, Germany; ^2^Leibniz Research Centre for Working Environment and Human Factors, >Dortmund, Germany; ^3^Department of Forensic Medicine and Toxicology, Faculty of Veterinary Medicine, South Valley University, Qena, Egypt; ^4^Electron Microscopy Facility, RWTH Aachen University Hospital, Aachen, Germany; ^5^Institute of Innate Immunity, University Hospital Bonn, Bonn, Germany; ^6^Department of Nephrology, The Royal Melbourne Hospital, Melbourne, VIC, Australia; ^7^Department of Medicine, University of Melbourne, Parkville, VIC, Australia

**Keywords:** calciprotein particle, calcification, inflammation, phosphate, fetuin-A, plasma protein

## Abstract

**Background:** The liver-derived plasma protein fetuin-A is a systemic inhibitor of ectopic calcification. Fetuin-A stabilizes saturated mineral solutions by forming colloidal protein-mineral complexes called calciprotein particles (CPP). CPP are initially spherical, amorphous and soft, and are referred to as primary CPP. These particles spontaneously convert into secondary CPP, which are larger, oblongate, more crystalline, and less soluble. CPP mediate excess mineral transport and clearance from circulation.

**Methods:** We studied by intravital two-photon microscopy the clearance of primary vs. secondary CPP by injecting i.v. synthetic fluorescent CPP in mice. We analyzed CPP organ distribution and identified CPP endocytosing cells by immunofluorescence. Cellular clearance was studied using bone marrow-derived mouse wildtype and scavenger receptor A (SRA)-deficient macrophages, as well as human umbilical cord endothelial cells (HUVEC), monocyte-derived macrophages (hMDM), and human aortic endothelial cells (haEC). We employed mouse wildtype and mutant immortalized macrophages to analyze CPP-induced inflammasome activation and cytokine secretion.

**Results:** In live mice, only primary CPP were rapidly cleared by liver sinusoidal endothelial cells (LSEC), whereas primary and secondary CPP were cleared by Kupffer cells. Scavenger receptor A (SRA)-deficient bone marrow macrophages endocytosed secondary CPP less well than did wildtype macrophages. In contrast, primary CPP endocytosis did not depend on the presence of SRA, suggesting involvement of an alternative clearance pathway. CPP triggered TLR4 dependent TNFα and IL-1β secretion in cultured macrophages. Calcium content-matched primary CPP caused twice more IL-1β secretion than did secondary CPP, which was associated with increased calcium-dependent inflammasome activation, suggesting that intracellular CPP dissolution and calcium overload may cause this inflammation.

**Conclusions:** Secondary CPP are endocytosed by macrophages in liver and spleen via SRA. In contrast, our results suggest that primary CPP are cleared by LSEC via an alternative pathway. CPP induced TLR4-dependent TNFα and inflammasome-dependent IL-1β secretion in macrophages suggesting that inflammation and calcification may be considered consequences of prolonged CPP presence and clearance.

## Introduction

Patients suffering from chronic kidney disease (CKD), especially patients on dialysis have exceedingly high cardiovascular morbidity and mortality (1). Apart from classical risk factors of cardiovascular disease (CVD) like smoking, hypertension, diabetes and dyslipidemia, elevated serum phosphate, and inflammation have been identified as major risk factors in these patients (2–4). These findings suggest that elevated circulating phosphate triggers CKD-associated CVD, and chronic inflammation and calcification amplify the detrimental effects of high serum phosphate, which cannot be properly regulated for lack of kidney function. Calcium and phosphate are required in millimolar concentrations for metabolism, signaling and hard tissue formation across many branches of the tree of life. In water, millimolar calcium and phosphate however, readily form insoluble salts unless the metastable state is stabilized by complex-forming compounds collectively referred to as mineralization inhibitors or mineral chaperones (5). This fundamental fact has been aptly named Lot's wife's problem (6). Vertebrates with bone made of calcium phosphate have evolved active inhibition mechanisms to prevent unwanted calcium phosphate deposition, calcification, outside the skeleton. The liver derived plasma protein fetuin-A is a systemic inhibitor of ectopic calcification. Fetuin-A deficient mice on a DBA/2 genetic background exhibit extensive extraosseous calcification, especially in brown adipose tissue, skin, heart, lung, and kidney (7, 8). Plasma fetuin-A is associated with and plays a critical role in the stabilization of protein-mineral complexes, named calciprotein particles (CPP) (9, 10). To prevent these calcifications, CPP first form as colloidal nanoparticles, containing fetuin-A, additional plasma proteins, and calcium and phosphate. Particles in this maturation state are called primary CPP. Primary CPP undergo Ostwald ripening with associated structural and compositional rearrangements (11). The resulting crystalloid secondary CPP are ellipsoid-shaped with a crystalline core surrounded by a protein layer (9). The protein layer is complex and shows marked differences in enrichment for certain proteins with particle ripening (12). Nonetheless, fetuin-A, albumin, and the plasma proteins apolipoprotein A1, prothrombin, and complement C3 have been consistently identified across multiple studies (13). Primary CPP reach a hydrodynamic diameter of roughly 50–100 nm, and secondary CPP measure 100–300 nm (11). Secondary CPP are cleared by macrophages via scavenger receptor A and thereby prevent pathological calcium and phosphate deposition. Liver Kupffer cells and marginal zone macrophages in the spleen were shown to be the main cell types involved in the clearance of secondary CPP (14). A mechanism for primary CPP clearance was never reported for lack of stable primary CPP preparations for use in animal and in cell-based studies.

Numerous studies showed that particle-induced inflammation causes cell damage both of the clearing macrophages and surrounding tissue cells alike by starting a vicious cycle of attempted clearance and chronic inflammation. Chronic inflammation is a salient part of e.g., ox-LDL clearance by atherosclerotic lesional macrophages, asbestos-induced inflammation in lung macrophages and indeed macrophage activation caused by a plethora of engineered nanoparticles. Crystalline particles like calcium-phosphate crystals induce an inflammatory response in cells, resulting in expression of TNF-α (15) and necrosis of the cells (16). Conflicting but not necessarily contradictory, data were reported on the inflammatory activity of calcium and phosphate, hydroxyapatite crystals, primary and secondary CPP. A side-by-side comparison in relevant cell types involved in clearance is missing. Multiple pathways have been implicated in the particle-induced secretion of inflammatory cytokines by e.g., clearing macrophages, which can be triggered by activation of the TLR4 (15), protein kinase C-α/activated protein kinase (17), or via the NLRP3 inflammasome (18, 19). Particles smaller than 1 μm and needle-shaped particles most potently induce an inflammatory response (20). The crystal-induced inflammatory response of macrophages can be markedly reduced by serum, which was partly attributed to the presence of fetuin-A in serum (21). Along these lines, fetuin-A containing secondary CPP had less inflammatory potential toward macrophages than had microcrystalline hydroxyapatite with respect to TNFα secretion (22). Thus, the formation of CPP from plasma proteins and calcium phosphate mineral both stabilizes the fluid phase in a metastable state, and dampens the inflammatory potential of mineral crystals, which might otherwise cause crystallopathies. It is however debatable, which form of CPP is more physiologically relevant. Primary CPP spontaneously form in biological fluids supersaturated with mineral ions, but the transformation into secondary CPP under biomimetic laboratory conditions can take many hours to days (23). The instability of primary CPP, which has greatly restricted previous studies, may also portend to spontaneous transformation *ex vivo*, thus at least a proportion of secondary CPP detected in some biological samples may represent a non-physiologically generated artifact. Indeed, we reported finding only copious amounts of secondary CPP in post-mortem ascites of a peritoneal dialysis patient who had died of sclerosing peritonitis (24). Other studies indicate that secondary CPP, while occasionally observed in serum and peritoneal effluent of dialysis patients, are present in much lower amounts than primary CPP (25). Thus, it remained of critical importance to understand the handling and effects of primary CPP in comparison with their more stable crystalline counterparts.

To this end we studied the clearance trajectories of primary and secondary CPP in live mice and in cultured cells. We report differential clearance of primary CPP and secondary CPP by liver sinusoidal endothelial cells (LSEC) and liver Kupffer cell macrophages, respectively. Furthermore, primary and secondary CPP differentially induced inflammatory responses in cultured macrophages. This finding is important for targeting the correct first responder cells, which may suffer phosphate or CPP-associated inflammatory damage *in vivo*.

## Materials and methods

### Animal experimentation

All animal experiments were conducted in agreement with the recommendation of the Federation for Laboratory Animal Science Associations (FELASA), and were approved by the animal welfare committee of the Landesamt für Natur-, Umwelt- und Verbraucherschutz (LANUV, 84-02.04.2013.A113 and 84.02.04.2015.A294). At least three mice each were injected in the clearance experiments and three mice of the genotypes were used to isolate bone marrow-derived macrophages. All mice were maintained in a temperature-controlled room on a 12-h day/night cycle. Food and water were given *ad libitum*.

### Protein purification and CPP preparation

Bovine fetuin-A (Sigma F2379) was purified by gel-filtration and labeled with Alexa-488 or Alexa-546 NHS ester (Thermo Scientific). Purified fetuin-A was routinely analyzed for LPS activity using the Endosafe ultrasensitive cartridge assay (Charles River). LPS content was <0.1 EU/ml, and did not induce cytokine secretion in macrophages. Labeled fetuin-A monomer was used to prepare primary and secondary CPP (14) or CPP were prepared in medium as described (26). Briefly, fetuin-A based CPP were produced in a solution containing 400 μl 2.5 mg/ml fetuin-A in 140 mM sodium chloride mixed with 100 μl 140 mM sodium chloride and 250 μl 24 mM phosphate buffer (pH 7.4). After thorough mixing, 250 μl of 40 mM calcium chloride solution (pH 7.4) was added. CPP formation proceeded at 37°C with all components pre-warmed. Primary CPP were harvested after 15 min incubation time and secondary CPP after 12 h. The DMEM/FCS-based CPP were prepared in pre-warmed solutions of DMEM containing 10% FCS. Phosphate and calcium were added as stock solutions of sodium phosphate (0.5 M, pH 7.4) and calcium chloride (0.5 M, pH 7.4) to final concentrations of 3.5 mM phosphate and 1.0 mM calcium, respectively. Primary CPP were collected after 1 day of incubation at 37°C and secondary CPP after 7 days. Particles were spun down at 20,000 × g for 20 min and afterwards resuspended in saline for further analysis.

### Particle analysis

Particle size distribution was analyzed using a NanoSight NS 300 particle analyzer (Malvern Instruments) equipped with a 488 nm laser module. Samples were diluted 1:100 in filtered sodium chloride solution (140 mM, passed through a 0.1 μm syringe filter). Depicted results are the average mode of 5 sequences of 30 s recording time each. For transmission electron microscopy, samples were diluted 1:10 in MilliQ water and 1 μl sample was applied onto Formvar-coated nickel grids (Plano, Wetzlar, Germany). The grids were dried at room temperature, and the CPP were visualized without staining. T_50_ assays of fetuin-A containing solutions for CPP preparations were performed as described (27) with minor modifications. Stock solution 1 was NaCl solution: 140 mM NaCl. Stock solution 2 was calcium solution: 40 mM CaCl_2_+100 mM Hepes+140 mM NaCl pH-adjusted with 10 M NaOH to 7.40 at 37°C. Stock solution 3 was phosphate solution: 19.44 mM Na_2_HPO_4_+4.56 mM NaH_2_PO_4_+100 mM Hepes+140 mM NaCl pH-adjusted with 10 M NaOH to 7.40 at 37°C. Before dispensing into 96-well plates, all solutions were prewarmed to 34.5°C. Liquid dispensing was done with a Liquidator96® bench-top pipetting system (Steinbrenner Laborsysteme GmbH) using fresh pipetting tips for every pipetting step. The components were mixed in the following order: (1) NaCl solution, 20 μl/well, (2) fetuin-A/BSA solution (1 mg/ml fetuin-A and 50 g/L BSA in 140 mM NaCl) or DMEM with 10% FCS, 80 μl/well, (3) phosphate solution, 50 μl/well, (4) mix 1 min, and (5) calcium solution, 50 μl/well, mix 1 min. The 96 wells were covered with ThinSeal adhesive sealing film. The assay was performed for 200 cycles with 1.5-s measurements per well and a position delay of 0.1 s in horizontal plate reading mode. Continuous measurements were recorded for hours or days as indicated in the figure legends.

### CPP clearance and intravital imaging

Functional intravital imaging of mouse livers and image analysis was performed as described (28) using a customized inverted microscope LSM MP7 (Zeiss, Jena, Germany) with an LD C-Apochromat 40x 1.1 water immersion objective. Briefly, the mice were anesthetized by an intraperitoneal injection of ketamine (100 mg/kg b.w), xylazine (10 mg/kg b.w.), acepromazin (1.7 mg/kg b.w.), and buprenorphine (0.08 mg/kg b.w.). Anesthesia was maintained throughout the observation period using an isoflurane inhaler. Mice were wiped with ethanol and their abdomen was shaved. The abdominal wall was opened with a transverse section of 1 cm length. The liver was carefully extraventralized by gravitational force without touching. The mouse with the liver protruding downward was mounted on a thermostated microscope stage using a large coverslip and pre-warmed Ringer saline. The mice received bolus intravenous injections of Hoechst 33258 and tetramethylrhodamine ethylester to allow visualization of nuclei and liver morphology, respectively (Table 1). At time zero, primary or secondary CPP were injected i.v. using a catheter inserted into the tail vein. Mice were injected with an equivalent of 160 μg (protein content) in a maximum volume of 200 μl of either monomeric fetuin-A, primary CPP, or secondary CPP, and intravital two-photon microscopy (2PM) videos were continuously recorded. All intravital imaging experiments were done in at least 3 mice.

**Table 1:** Fluorescent marker dyes and imaging conditions.

**Fluorescent marker dye**	**Marker for**	**Dose [mg/kg b.w.]/vehicle**	**Excitation range [nm]**	**Source**
Hoechst 33258	Nuclei	5 in PBS	720–800	Thermo scientific
Tetramethylrhodamine ethylester (TMRE)	Mitochondrial membrane potential	0.96 in methanol: PBS (1:1)	780–820	Thermo scientific
Alexa-488-labeled fetuin-A	Primary/Secondary CPP	160 μg (protein) in 0.9% NaCl	740–800	Thermo scientific / Sigma

### Organ preparation and immunofluorescence

In a separate set of injection experiments mice were anesthesized and injected as above, and sacrificed at times indicated in the figure legends by isofluorane overdosing and organs were collected for histology. Tissues were embedded in Tissue-Tek O.C.T. compound to prepare 5 μm cryosections for subsequent CPP distribution analysis and immunofluorescent staining. Sections were fixed for 5 min with Bouin's fixative, rinsed with 0.1 M Tris pH 8.5, washed with distilled water and blocked with 10% goat serum in PBS. Primary antibodies against F4/80 (ab6640, Abcam) and LYVE-1 (DP3513P, Acris) were used to stain macrophages and liver sinusoidal endothelial cells, respectively. Secondary antibodies were Alexa-488 or Alexa-546 labeled goat anti-rat antibodies (A11006, A11081, Thermo Fisher Scientific). After antibody staining, sections were counterstained with DAPI and mounted in Immu-Mount (Thermo Fisher Scientific). Micrographs were recorded using a Leica DM-IRB 6000 inverted microscope, appropriate illumination and filter sets and a high sensitivity digital Leica black and white camera. Fluorescence micrographs were color-coded and compound fluorescence pictures were composed and enhanced using Adobe Photoshop software. Photos in each figure plate were processed identically.

### Cell culture experiments

Murine immortalized wildtype and TLR4 knockout macrophages (29) were cultured in RPMI supplemented with 10% fetal calf serum (FCS). Primary bone marrow-derived macrophages were isolated from C57BL/6 wildtype or SRA-deficient mice (14). The cells were differentiated for 10 days using L929-fibroblast conditioned medium. HUVEC were cultured in EGM-2 (Lonza). For endocytosis assays, primary wildtype and SRA-knockout bone marrow-derived macrophages prepared from at least three mice each (14) or human umbilical cord venous endothelial cells HUVEC were seeded in 0.5 ml medium at 500,000 cells/ml in 24-well plates. After overnight culture the cells were incubated for 1 h at 37°C with 100 μg (protein content) in 500 μl medium Alexa488-labeled or Alexa546-labeled fetuin-A monomer. The same amount of labeled fetuin-A was contained in primary or secondary CPP as indicated in the figure legends. Cells were washed twice with PBS, and observed under a microscope, or fixed with 4% paraformaldehyde and fluorescent CPP uptake was measured by flow cytometry. To assess inflammatory cytokine release, immortalized wildtype and TLR4 knockout (ko) macrophages were seeded at 250,000 cells/ml in 24-well plates and kept overnight. For TNFα measurement, cells were starved for 2 h in serum-free RPMI, and then treated with 200 μg/ml (calcium content) primary or secondary CPP, or with 10 ng/ml LPS. After 6 h incubation the culture supernatants were harvested and TNFα levels were measured by ELISA (DY410-05, R&D systems). For IL-1β measurement, cells were primed with 10 ng/ml LPS for 2 h, and subsequently treated with primary or secondary CPP, or with 10 ng/ml LPS for 16 h. Cell culture supernatant was harvested and secreted IL-1β was determined by ELISA (DY401-05, R&D systems).

To test the clearance of primary and secondary CPP in human cells, human monocyte-derived macrophages (hMDM) and primary human aortic endothelial cells (haEC) were also studied. Alexa488-labeled bovine fetuin-A was spiked into 10% FCS/DMEM culture medium to synthesize primary and secondary CPP as described previously (26). Particle synthesis was confirmed by cryoTEM. Monomeric labeled-fetuin-A (1 mg/ml) was also run alongside as a comparator. hMDM were cultured as described (30) with minor modifications. In brief, peripheral blood mononuclear cells were isolated from the buffy coats (diluted 1:1 in PBS containing 2 mM EDTA; PBS-EDTA) of freshly drawn blood of healthy volunteers by Ficoll-paque (GE Healthcare) density centrifugation (350 g for 30 min at 4°C). The mononuclear cell layer was recovered and washed twice in PBS-EDTA and then enriched for monocytes by positive selection with anti-CD14 magnetic beads (Miltenyi Biotec). Macrophages were obtained from these isolates after 7 days of culture in macrophage serum-free medium (Invitrogen, Carlsbad, CA, USA) supplemented with 10% (vol/vol) human serum (Sigma, St. Louis, MS, USA), which was cleared from cryoprecipitates and other particulates/exosomes by centrifugation (18 h at 350,000 × g and 4°C). The medium also contained human macrophage colony-stimulating factor (50 ng/ml; eBioscience, San Diego, CA, USA), penicillin (100 U/ml; Sigma), and streptomycin (0.1 mg/ml; Sigma). Cells were cultured at 37°C and an atmosphere of 5% CO_2_. haEC were purchased from Lonza (#CC-2535; Walkersville, USA), cultured in EGM-2 BulletKit (#CC-3162; Lonza) to 70–80% confluence and used at passage 3–7.

For endocytosis assays, cells were seeded in 24-well plates (250,000 cells per well) and rested overnight in fresh culture media prior to experimentation the next day. Cultures were treated in serum-free media supplemented with Alexa488-labeled primary CPP or secondary CPP adjusted to equivalent levels of calcium (100 μg/ml), or monomeric labeled-fetuin-A (1 mg/ml) for 10, 20, 60, or 120 min, after which monolayers were gently washed and fixed with BD Phosflow Fix buffer I (containing 4.2% formaldehyde). Cell-associated fluorescence was measured by flow cytometry using the 488 nm laser for excitation and the FITC filter (527/32) for detection. Non-linear functions were used to fit averaged data for each treatment. In some experiments cells were co-treated with both primary and secondary CPP to investigate competitive uptake. While in others, cells were pre-treated for 30 min with one of several chemical inhibitors at previously defined concentrations as detailed in Supplementary Table 1 (all from Sigma): cytochalasin D (10 μM), chlorpromazine (10 μg/ml), filipin (2 μg/ml), genestein (200 μM), 5-(N,N-dimethyl)amiloride hydrochloride (10 μM; 5-DMA), MβCD (2 mM), Ly294002 (100 μM), monodansylcadaverine (100 μM; MDC) polyinosinic acid (10 μg/ml) or vehicle (DMSO/media); then washed and switched to media containing AF488-CPP (100 μg/ml) and incubated for 60 min at 37°C; or were pre-treated with blocking antibodies (60 min at 4°C) directed against SRA (AbD Serotec, 10 μg/ml;), TLR2/4/6 (10 μg/ml; Invivogen), CD36 (clone FA6-152; 10 μg/ml; Hycult Biotech), or annexin-2 (20 μg/ml; BD Biosciences) or relevant isotype IgG control (all 10 μg/ml; eBioscience) before incubation with CPP-containing media for 60 min at 37°C.

### Statistics

All statistical analyses were carried out using GraphPad Prism version 5.0c and are given as mean values ± standard deviations. One-way ANOVA with Tukey's multiple comparison test was used to test for differences in non-sized matched experimental groups.

## Results

### Particle morphology is independent of synthesis route

**Figure 1:**
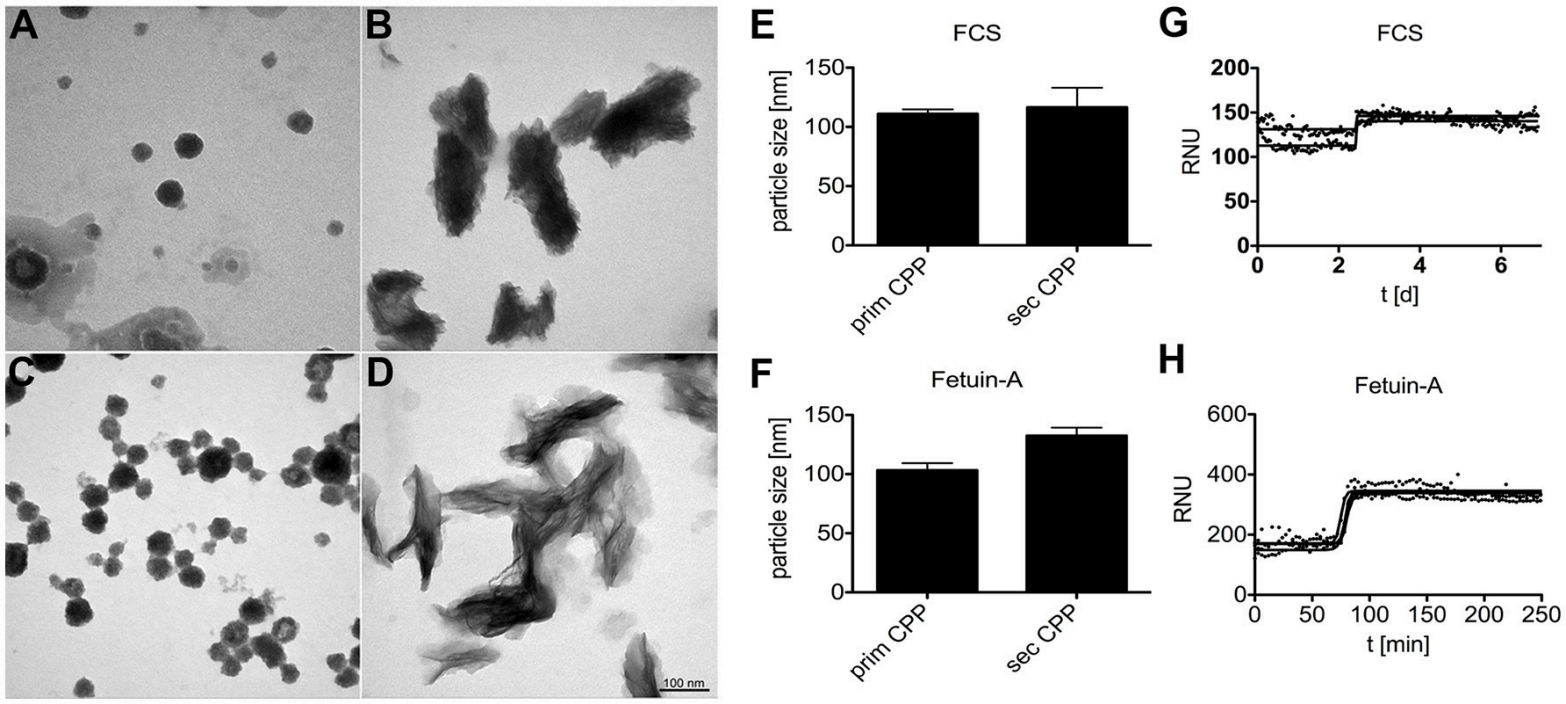
Calciprotein particle CPP formation in protein solutions supersaturated with calcium and phosphate. **(A,B,E,G)** CPP were prepared in DMEM supplemented with 1.0 mM calcium (total Ca 3.8 mM), 3.5 mM phosphate (total Pi 4.4 mM), and 10% fetal calf serum FCS. **(C, D,F,H)** Alternatively, CPP were prepared in HEPES/NaCl buffer supplemented with 10 mM calcium, 6 mM phosphate, and 1.0 mg/ml purified fetuin-A protein. **(A–D)** CPP were harvested by centrifugation and analyzed by electron microscopy or by nanoparticle tracking analysis **(E,F)**. **(A–D)** Depict representative CPP in their primary **(A,C)** and secondary **(B,D)** state. DMEM/FCS-derived and HEPES/fetuin-A-derived primary CPP were similar, but DMEM/FCS-derived secondary CPP appeared denser, yet less crystalline because of higher protein content. Electron microscopy shows that primary and secondary CPP are distinct in shape and size, while nanoparticle tracking analysis **(E,F)** indicated similar particle sizes of around 100–125 nm for all preparations. Continuous nephelometer measurements **(G,H)** indicated a sharp transformation of primary into secondary CPP around 2.5 days and 75 min in the presence of DMEM/FCS and HEPES/fetuin-A protein, respectively.

We analyzed by electron microscopy the supernatant of a buffered solution supersaturated in calcium and phosphate. The solution also contained purified fetuin-A protein or FCS, which is rich in fetuin-A protein. Both solutions mediate the formation of protein-mineral complexes known as calciprotein particles (CPP). Figures 1A–D show primary and secondary CPP formed in the presence of FCS (Figures 1A,B) or in the presence of purified fetuin-A protein (Figures 1C,D). Primary CPP appeared as spherical particles with a size 50–100 nm in diameter (Figures 1A,C). Secondary CPP had overall elliptic shape of up to 100 nm wide and up to 250 nm long. Crystalline spicules were observed in secondary CPP, but not in primary CPP. Nanoparticle tracking analysis measurements shown in Figures 1E,F indicated particles with hydrodynamic diameters of 100–125 nm in all preparations, indicating a strong bias of this method toward the shorter particle axis. Importantly, primary and secondary CPP each were morphologically indistinguishable regardless of their route of synthesis. Overall our findings thus confirm numerous studies by our and other laboratories indicating that spherical, poorly crystalline primary CPP spontaneously formed immediately after mixing supersaturated solutions of calcium and phosphate in the presence of purified fetuin-A or in the presence of serum containing fetuin-A; and that the primary CPP spontaneously transformed into elongated secondary CPP of about twice the length. Figures 1G,H illustrate that the characteristic Ostwald transformation of primary CPP into secondary CPP occurred irrespective of the mineral supersaturation of the mineral solutions, or the protein content, albeit at widely varying times. We derived computationally from continuous light scatter measurements the crystal transformation time (T_50_), which indicates the half-maximum transformation of primary into secondary CPP. T_50_ was 2.5 days if CPP formed in DMEM supplemented with 1 mM Ca (total Ca 3.8 mM), 3.5 mM phosphate (total Pi 4.4 mM), and 10% fetal calf serum FCS (Figure 1G). T_50_ dropped to 350 min in the presence of 10% FCS (not shown), and further to 75 min in the presence of 1 mg/ml purified fetuin-A (Figure 1H), both dissolved in HEPES/NaCl buffer supplemented with 10 mM calcium, 6 mM phosphate at neutral pH. Thus, protein concentration was positively associated with T_50_, while mineral ion supersaturation was negatively associated with T_50_. We employed primary and secondary CPP formed with or without fluorescence labeled fetuin-A as indicated in the figure legends, to study their clearance from circulation in live mice, and to study CPP biological activity in cultured macrophages.

### Fast clearance of primary and secondary CPP in the liver

**Figure 2:**
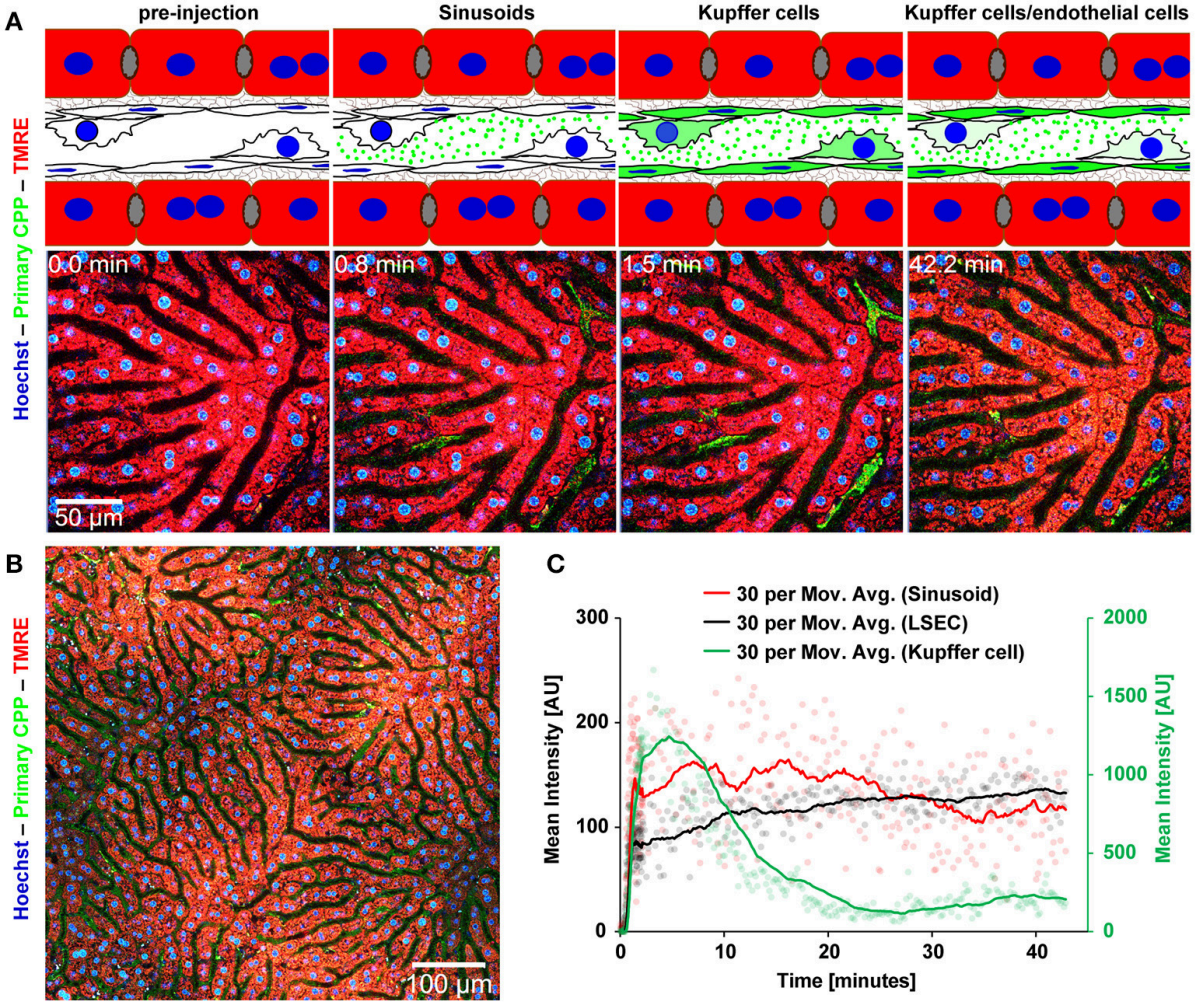
Functional imaging of primary CPP clearance using 2-photon intravital microscopy. Mice received intravenous injections of primary CPP prepared with fluorescent fetuin-A, and the major clearance organ liver was continuously recorded for up to 1 h. Fluorescent intensity was quantified in regions of interest (ROI) and clearance kinetics were calculated. Representative still micrographs with typical views of primary CPP clearing cells are shown. **(A)** Primary CPP clearance from sinusoids into liver sinusoidal endothelial cells (LSEC) occurred within 1 min, and into Kupffer cells within 2 min. Thereafter, primary CPP in Kupffer cells were rapidly degraded, shown by a fast decline of the signal after about 15 min. **(B)** Overview showing a sector of a liver lobule. **(C)** Calculation of mean fluorescence intensity in the sinusoids, LSEC, and Kupffer cells determined by ROI quantification.

**Figure 3:**
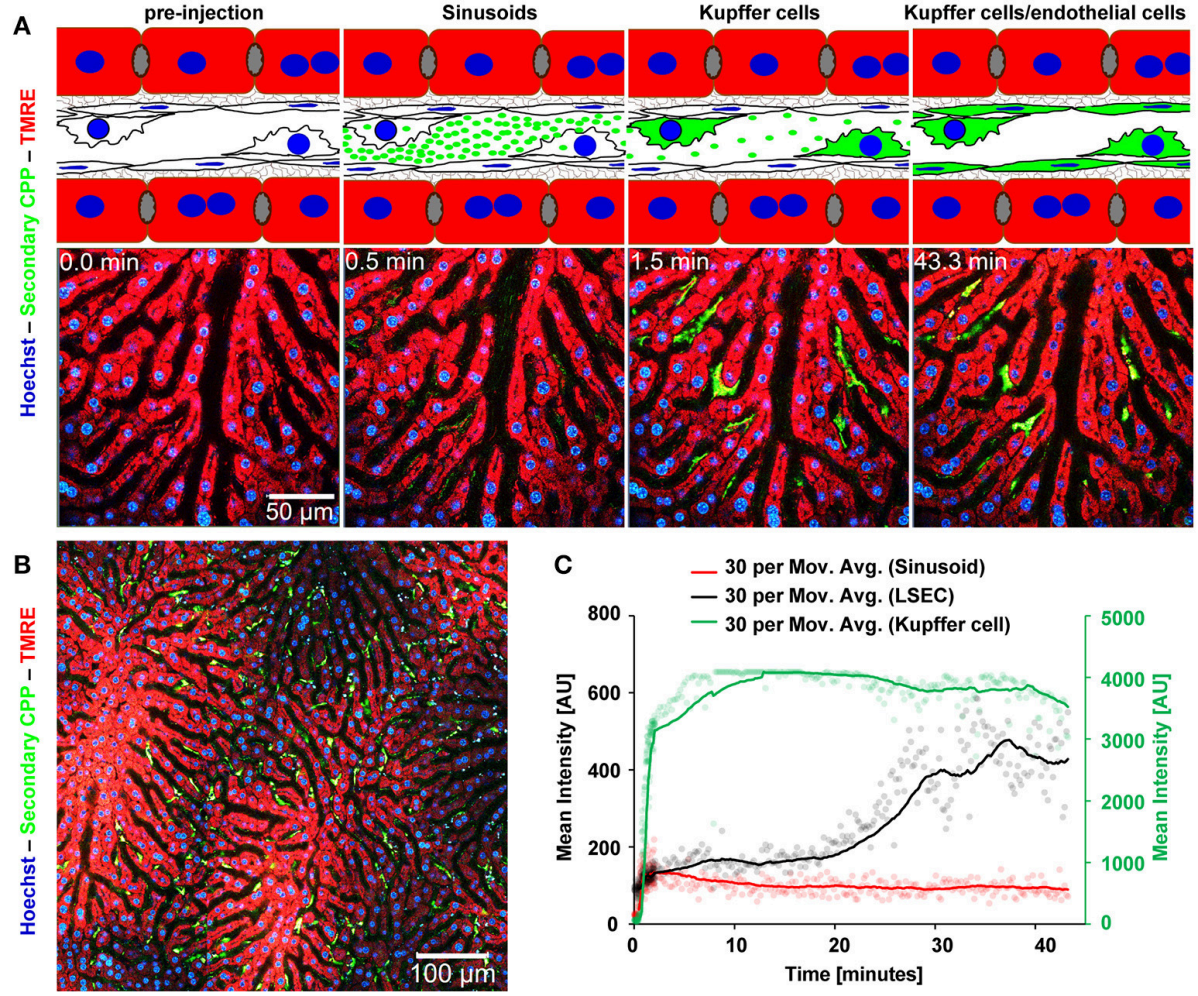
Functional imaging of secondary CPP clearance using 2-photon intravital microscopy. Mice received intravenous injections of secondary CPP prepared with fluorescent fetuin-A, and the major clearance organ liver was continuously recorded for up to 1 h. Fluorescent intensity was quantified in regions of interest (ROI) and clearance kinetics were calculated. Representative still micrographs with typical views of secondary CPP clearing cells are shown. **(A)** Secondary CPP clearance from sinusoids, first into Kupffer cells occurred within 2 min and further into liver sinusoidal endothelial cells (LSEC) within 25 min as illustrated by the cartoons above the micrographs. No decline in Kupffer cell associated fluorescence was determined during the observation period. **(B)** Overview showing a sector of a liver lobule. **(C)** Calculation of mean fluorescence intensity in the sinusoids, LSEC, and Kupffer cells determined by ROI quantification.

Mice received intravenous bolus injections of 160 μg (fetuin-A content, equals 160 μg calcium content) of fluorescent primary or secondary CPP, an amount which should rapidly be diluted to 10 μg/ml CPP (calcium content) or below in the circulation, a concentration of circulating CPP recently observed in CKD patients (25). We studied CPP liver clearance by intravital 2-photon microscopy. Figure 2A shows that primary CPP clearance from sinusoids into liver sinusoidal endothelial cells (LSEC) occurred in <1 min. Since the sinusoidal endothelial cells are typically thin, the CPP associated signal is difficult to see on the stills of Figure 2A and can more easily be studied in the videos (Supplementary Movie 1). Kupffer cells showed a strong CPP associated fluorescence as early as 2 min after injection. Thereafter, primary CPP in Kupffer cells were rapidly degraded, shown by a fast decline in signal after about 15 min. Figure 3 illustrates secondary CPP clearance from sinusoids, first into Kupffer cells within 2 min, and only later ~25 min after injection into LSEC as illustrated by the cartoons above the micrographs. Unlike in primary CPP, no decline in Kupffer cell-associated fluorescence was observed with secondary CPP during the observation period. Kupffer cell-associated fluorescence following secondary CPP clearance was three- to four-fold higher than sinusoidal endothelial cell-associated fluorescence following primary CPP suggesting preferential clearance of crystalloid particles like secondary CPP over soft colloidal particles like primary CPP. Supplementary Movies 1, 2 provided in the supplement illustrate complete clearance sequences of primary and secondary CPP in the liver underscoring differential clearance by LSEC and Kupffer cell macrophages, respectively.

### Liver sinusoidal endothelial cells predominantly clear primary CPP, liver kupffer cells predominantly clear secondary CPP

**Figure 4:**
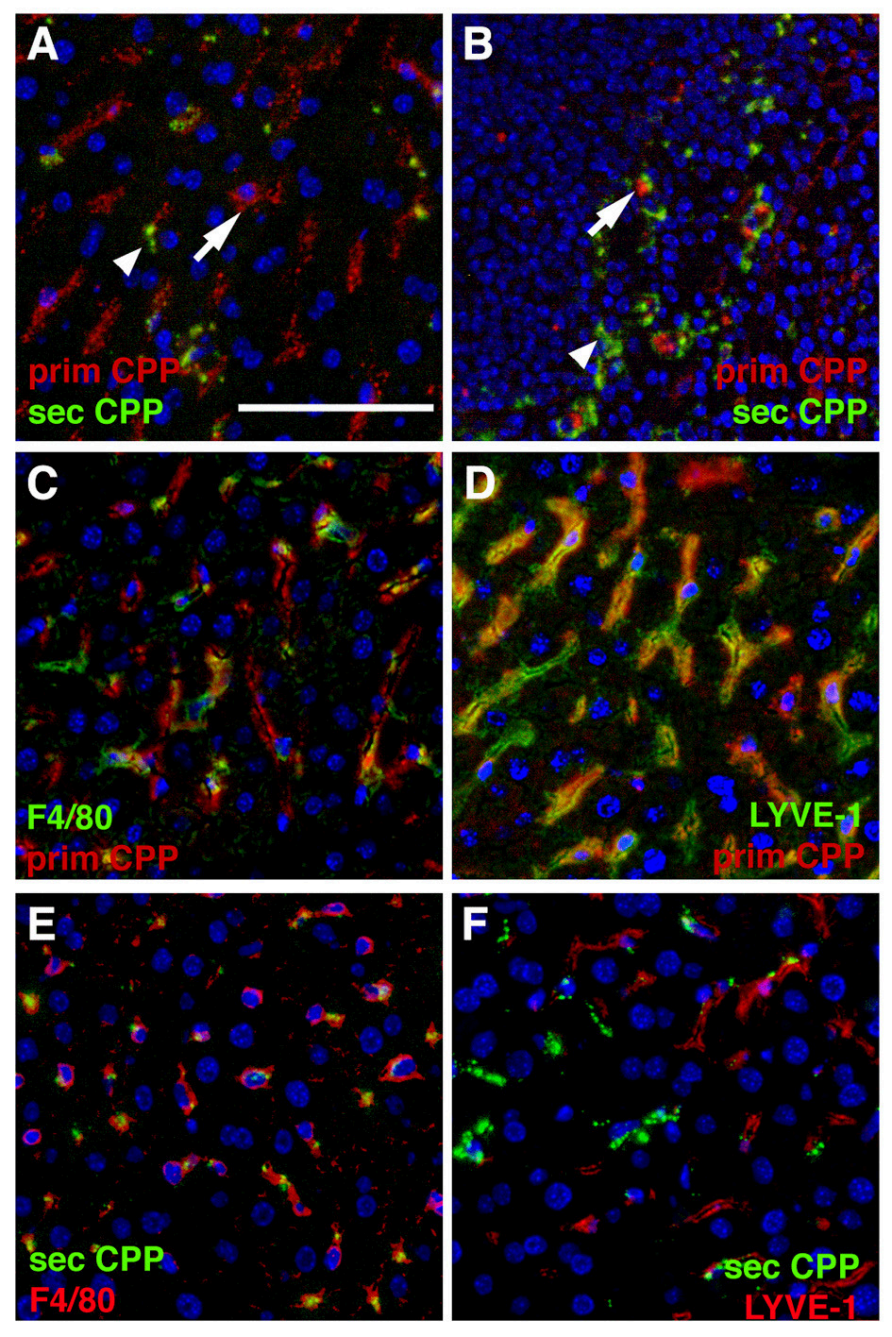
Differential clearance of primary and secondary CPP. Mice were injected with a mixture of fluorescence labeled primary (red) and secondary CPP (green) and the major clearance organs liver **(A,C–F)** and spleen **(B)** were harvested 10 min after injection, sectioned and analyzed for the presence of CPP. Primary CPP (prim CPP, arrows in **A,B**) and secondary CPP (sec CPP, arrow heads in **A,B**) showed distinct non-overlapping distribution in liver **(A)**, and spleen **(B)**. **(C–F)** Co-localization with the macrophage-specific marker F4/80 and the liver sinusoidal endothelial LSEC-specific marker LYVE-1 suggested that primary CPP were predominantly cleared by LYVE-1-positive LSEC, and secondary CPP were predominantly cleared by F4/80-positive liver Kupffer cell macrophages. Scale bar: 25 μm.

In a separate series of experiments we injected mice with a mixture of Alexa546-labeled primary (red) and Alexa488-labeled (green) secondary CPP, harvested organs as indicated in the figure legends, and analyzed CPP distribution in two major clearance organs of the reticulo-endothelial system, liver and spleen. Like in the intravital clearance experiments shown in Figures 2, 3, we observed little overlap in the cell types involved in the clearance of primary CPP (arrows in Figures 4A,B) and secondary CPP (arrow heads in Figures 4A,B), both in liver (Figure 4A), and spleen (Figure 4B). Unlike liver and spleen, pancreas, kidney, lung, myocard, and brown adipose fat tissue (BAT) cleared neither primary nor secondary CPP (Supplementary Figure 3). We focused on CPP clearance in the liver, because this organ has much higher mass than spleen and will clear most CPP despite similar density of clearing cells (Figures 4A,B, Supplementary Figure 3). We positively identified the clearing cell types employing immunostaining for the Kupffer cell macrophage marker F4/80 (Figures 4C,E). Alternatively, we stained for the liver sinusoidal endothelial cell (LSEC) marker LYVE-1 (Figures 4D,F). Figures 4C,D illustrate that primary CPP clearance was predominantly mediated by LYVE-1-positive LSEC, implicating for the first time the involvement of a non-myeloid cell type in the clearance of circulating protein-mineral complexes. In contrast, secondary CPP were predominantly cleared by F4/80-positive Kupffer cells (Figures 4E,F) corroborating our previous report (14). The finding that primary CPP were cleared by LSEC, while secondary CPP were cleared by liver Kupffer cells is important, because recent research has indicated that early stage protein mineral complexes like primary CPP are likely involved in the pathology of chronic kidney disease-associated calcification, while secondary CPP may indicate prolonged non-physiological ripening of protein-mineral complexes (4).

### Scavenger receptor a-mediated endocytosis and differential clearance of CPP in macrophages and endothelial cells

**Figure 5:**
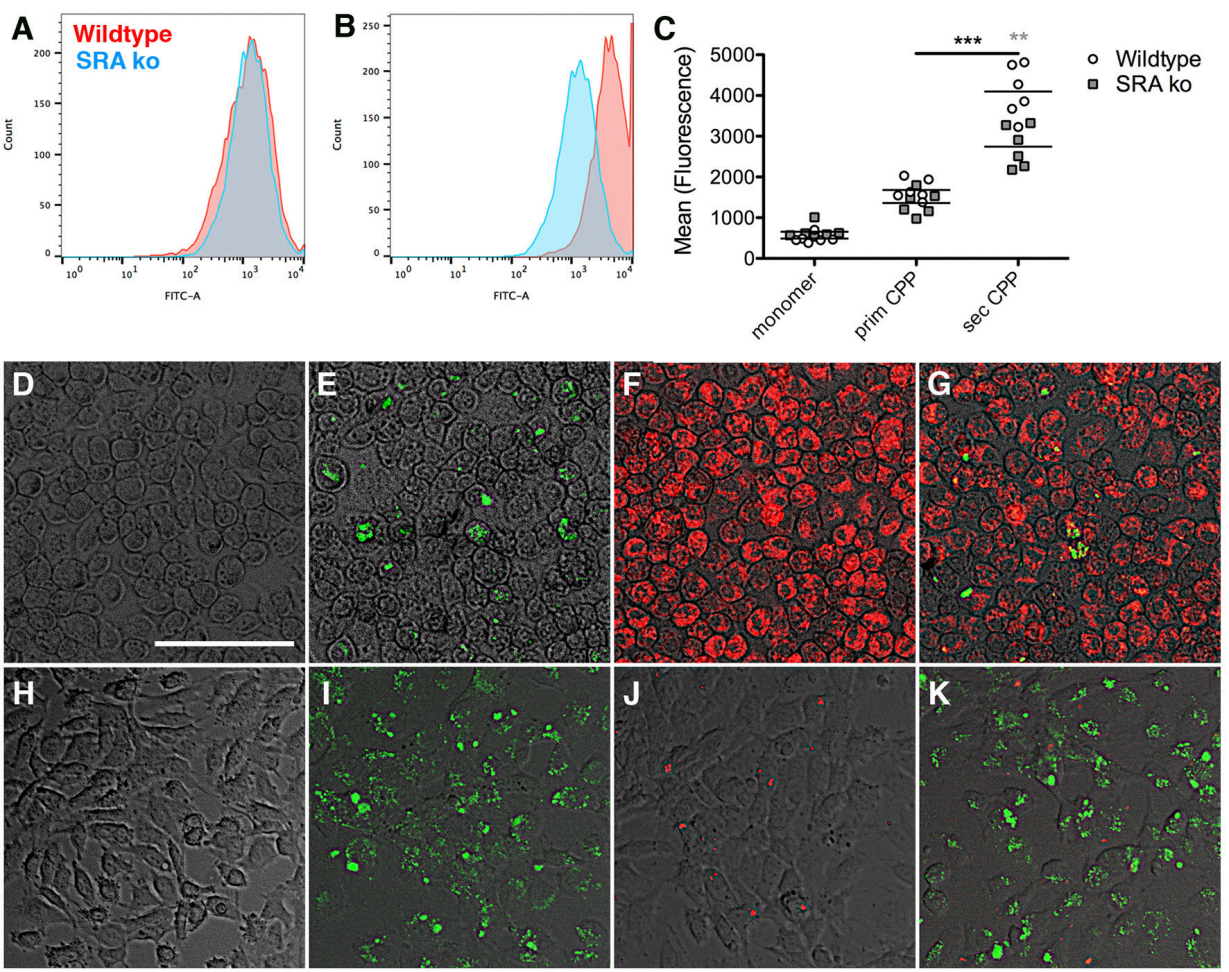
Differential endocytosis of primary and secondary CPP in mouse macrophages and endothelial cells. **(A–C)** Primary bone marrow macrophages derived from wildtype and scavenger receptor A-deficient mice (SRA ko) were treated with fluorescence labeled fetuin-A monomer (not shown) or **(A)**, primary or **(B)**, secondary CPP for 1 h, and endocytosis was analyzed by flow cytometry. **(C)** Quantitation of endocytosis shows negligible fetuin-A monomer endocytosis, low primary CPP endocytosis but strong secondary CPP endocytosis, which was reduced in SRA-deficient macrophages vs. wildtype. **(D–G)** Fluorescence micrographs of wildtype macrophages and **(H–K)**, HUVEC cells treated for 1 h with **(D,H)** labeled fetuin-A monomer **(E,I)** labeled primary CPP (green fluorescence), **(F,J)** secondary CPP (red fluorescence), or **(G,K)** a mixture of primary and secondary CPP. Macrophages endocytosed predominantly secondary CPP **(E–G)**, but HUVEC endocytosed predominantly primary CPP **(I–K)**, regardless of whether CPP were incubated individually **(E,F,I,J)** or in combination **(G,K)**. Neither cell type endocytosed detectable amounts of fetuin-A monomer **(D,H)**. Scale bar in **(D)** 75 μm. **p < 0.01, ***p < 0.001.

Previously we showed that macrophage clearance of secondary CPP was predominantly mediated by scavenger receptor A (SRA) (14). Here we asked if primary CPP clearance likewise required SRA. To this end we treated primary bone marrow macrophages derived from wildtype and scavenger receptor A-deficient mice (SRA ko) for 1 h with fluorescence-labeled fetuin-A monomer, fluorescent primary or secondary CPP, and analyzed endocytosis by flow cytometry. To discriminate fetuin-A and CPP binding from endocytosis we incubated bone marrow-derived macrophages with 100 μg (protein content) in 500 μl medium of fetuin-A monomer, primary and secondary CPP at 4°C to assess binding, or at 37°C to assess endocytosis. Following 1 h of incubation we analyzed cell-associated fluorescence by flow cytometry. Incubation at 4°C yielded much lower cell-associated fluorescence than incubation at 37°C, especially for primary and secondary CPP (Supplementary Figure 4). These results indicate that the cells readily endocytosed secondary CPP, and less well also primary CPP. A small amount of fetuin-A monomer was also endocytosed. Figure 5A shows that wildtype and SRA ko macrophages endocytosed small amounts of primary CPP equally well. Figure 5B shows that the macrophages endocytosed five-fold larger amounts of secondary CPP confirming the results shown in Supplementary Figures 3, 4, and mirroring the Kupffer cell discrimination of secondary CPP illustrated in Figure 3C. However, SRA-deficient macrophages endocytosed secondary CPP roughly 40% less efficient than did wildtype macrophages corroborating SRA is a major endocytosis receptor for secondary CPP as reported (14), but not for primary CPP. Figures 5D–G illustrate the strong discrimination of CPP endocytosis in macrophages, which endocytosed no detectable fetuin-A monomer (Figure 5D), small amounts of primary CPP (Figures 5E,G, green fluorescence), but large amounts of secondary CPP (Figures 5F,G red fluorescence). This strong differential endocytosis of primary vs. secondary CPP was maintained when the macrophages were treated simultaneously with primary and secondary CPP, indicating that the endocytic pathways are separate and non-competitive (Figure 5G). Primary vs. secondary CPP discrimination was reversed in HUVEC. Like macrophages, HUVEC endocytosed no detectable fetuin-A monomer (Figure 5H), yet unlike macrophages endocytosed large amounts of primary CPP (Figures 5I,K, green fluorescence), but small amounts secondary CPP (Figures 5J,K red fluorescence). Like in macrophages, HUVEC discrimination of primary vs. secondary CPP was maintained when the cells were treated simultaneously with primary and secondary CPP (Figure 5K).

**Figure 6:**
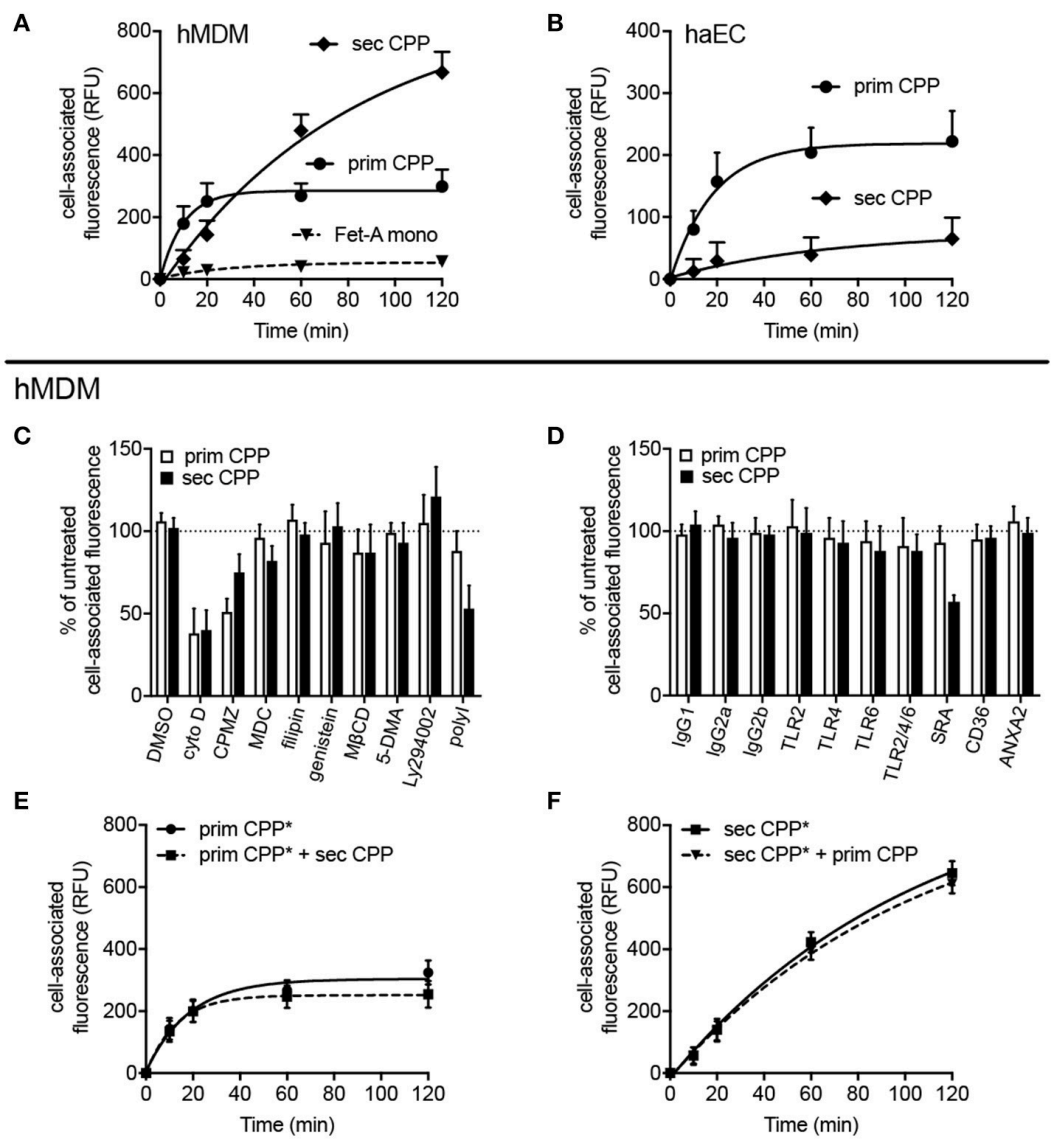
Differential endocytosis of primary and secondary CPP in human monocyte-derived macrophages and aortic endothelial cells. Human monocyte-derived macrophages (hMDM) or human aortic endothelial cells (haEC) were treated with Alexa488-labeled primary or secondary CPP, both 100 μg/mL calcium content, synthesized from FCS/DMEM supplemented with phosphate (final conc. 3.5 mM). Monomeric labeled-fetuin-A was included as a comparator (1 mg/ml). Cell-associated fluorescence was measured by flow cytometry in fixed cells at the stated timepoints. **(A)** Endocyosis observed in hMDM and **(B)** haEC, respectively. **(C,D)** hMDM were pre-treated for 30–60 min with one of several inhibitors (vs. vehicle) or receptor blocking antibodies (vs. isotype control). **(C)** Pre-treatment with cytochalasin **(D)**, an inhibitor of actin polymerization, markedly attenuated uptake of both primary and secondary CPP and (<50% untreated signal; *p* < 0.01) indicating the requirement for active actin-mediated cytoskeletal rearrangement for CPP internalization. Inhibition of protein tyrosine kinase/caveolin-dependent endocytosis (filipin and genestein), lipid-raft synthesis (methyl cyclodextrin-β-cyclodextrin; MβCD), macropinocytosis [5-(N,N-dimethyl)amiloride; 5-DMA], and phosphoinositol-3-kinase-dependent Fcγ receptor-mediated endocytosis (Ly294002) all had no significant effect on CPP uptake. Likewise, pre-treatment with monodansylcadaverine (MDC), an inhibitor of clathrin-mediated endocytosis had no effect on particle uptake. Inhibition of clathrin-dependent endocytosis with chlorpromazine significantly reduced primary CPP internalization (<50%; *p* < 0.05) but not secondary CPP, but may potentially reflect off-target effects due to the non-specificity of the inhibitor. **(D)** Specific immunochemical blockade of TLR2, TLR4, and TLR6, independently of one another, or in combination failed to significantly inhibit uptake of primary or secondary CPP. Neither blockade of CD36, a class B scavenger receptor involved in the uptake of modified LDL particles, or annexin 2, a putative receptor for fetuin-A cellular attachment, had a significant effect on CPP internalization. Consistent with data in the murine macrophage, competitive chemical inhibition of the SR-A with polyinosinic acid (polyI) or immunochemical blockade, markedly decreased endocytosis of secondary CPP (>50%, *p* < 0.001), but not primary CPP. **(E,F)** labeled primary or secondary CPP (CPP*) were added to hMDM with a four-fold excess of unlabeled secondary or primary CPP, respectively to assess whether uptake was competitive. Data are expressed as the mean ±SD, from 6 **(A,B,E,F)** or 4 **(C,D)** independent experiments.

To test the clearance of CPP in primary human cells cultures, human monocyte-derived macrophages (hMDM) and human aortic endothelial cells (haEC) were also studied. Figures 6A,B show that hMDM—like mouse macrophages—predominantly cleared secondary CPP, while haEC—like mouse LSEC and HUVEC—predominantly cleared primary CPP. Figures 6C,D show that the endocytosis inhibitors cytochalasin D (cyto D), chlorpromazine (CPMZ), and polyinosinic acid (polyI) partially inhibited CPP endocytosis suggesting vesicle-mediated endocytosis of both kinds of CPP, and the involvement of scavenger receptor A (blocked by polyI) exclusively in the endocytosis of secondary CPP, confirming the results shown in Figure 5 using SRA ko mouse macrophages. Antibody blockade of various receptors confirmed this finding in that anti-SRA antibodies inhibited secondary CPP endocytosis, but not primary CPP endocytosis in hMDM. Figures 6E,F show that neither primary nor secondary CPP uptake by hMDM was competitive, again suggesting involvement of distinct cell surface receptors. The fact that both primary and secondary CPP uptake was strongly reduced upon cytoD treatment and partially reduced upon chlorpromazine treatment, albeit more so for primary than secondary CPP, suggested similar endocytic trajectories once the particles had bound their cell surface receptors and endocytosis had begun.

### CPP induced inflammatory cytokine secretion requires inflammasome activation and TLR4-interaction

**Figure 7:**
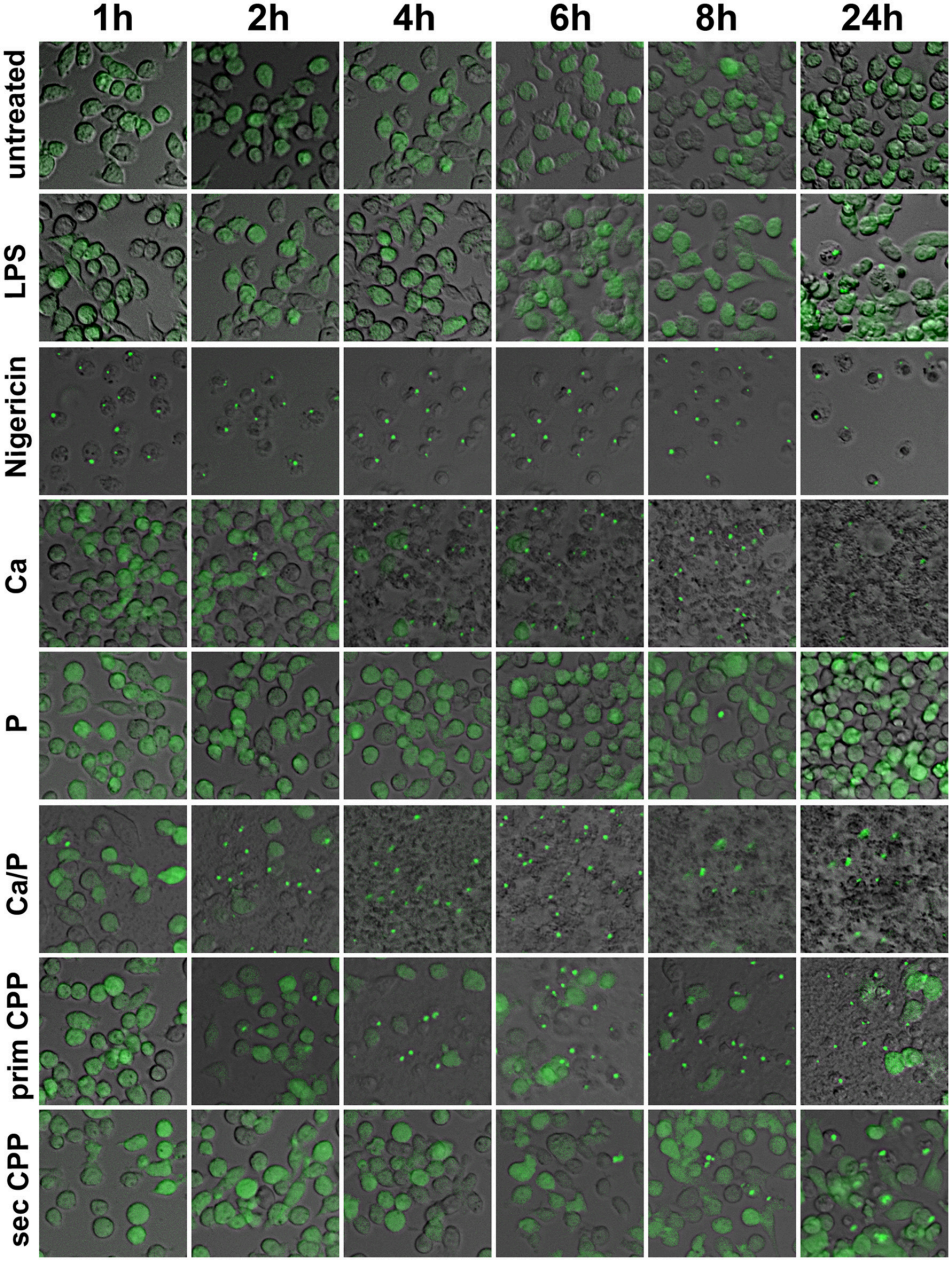
Calcification media induce differential cellular calcium overload and inflammasome activation. Immortalized macrophages expressing the inflammasome adaptor protein labeled *apoptosis-associated speck like protein containing a caspase recruitment domain* ASC fused to *green fluorescent protein*, were exposed to buffer control, LPS, nigericin, or to calcification media with elevated calcium or phosphate, individually and in combination, as well as with primary or secondary CPP. Notably, all calcification media contained an identical total calcium dose of 100 μg, yet differed in the stability of the calcium containing mineral component.

Next we asked if calcification media components and CPP endocytosis in particular, triggered an inflammatory response in macrophages and if so, which molecular pathways were involved. We reasoned that the particle nature of CPP might trigger inflammasome activation. To this end we performed speck formation assays using immortalized macrophages expressing the inflammasome adaptor protein *apoptosis-associated speck like protein containing a caspase recruitment domain* fused to *green fluorescent protein* (ASC-GFP). These macrophages are endogenously primed for inflammasome activation (31). We treated the ASC-GFP macrophages with either buffer control, LPS, nigericin, or with calcification media containing elevated calcium or phosphate, or both, as well as with primary or secondary CPP. All calcification media contained identical total calcium as indicated in the figure legends. Figure 7 shows micrographs of ASC-GFP macrophages with fluorescent specks signifying the recruitment of cytoplasmic ASC into the inflammasome. Of all treatments, nigericin caused the most rapid inflammasome activation, which was accompanied by loss of cellular integrity and cell detachment. In decreasing order of inflammasome activation and deteriorating cell integrity, Ca, Ca + phosphate, primary CPP, secondary CPP, and LPS all caused inflammasome recruitment of ASC and the ensuing cell loss. The late activation by LPS alone was expected, because LPS is known to merely prime macrophages for a “second hit” that will trigger definitive inflammasome activation. The lack of inflammasome activation by elevated phosphate suggests that the phosphate-triggered inflammatory cell activation and ultimately also calcification reported in many studies is either independent of inflammasome activation or is in fact caused by spontaneous calcium phosphate precipitation in the cell culture medium that went unnoticed. Figure 7 strongly suggests that all Ca-containing preparations triggered inflammasome activation in the order of solubility and thus in the order of availability of ionized Ca to stimulate the macrophages by cellular calcium overload. Nigericin caused immediate influx of cytoplasmic Ca and hence the fastest and strongest cytoplasmic Ca stimulation. Elevated extracellular Ca added to the culture medium alone or in combination with elevated phosphate caused similar intermediate inflammasome activation and cell damage. Primary CPP and secondary CPP caused intermediate to mild inflammasome activation, respectively, accompanied by corresponding reduced cell damage. Collectively, the results presented in Figure 7 suggest that cytoplasmic calcium is the strongest trigger of speck formation and that calcium phosphates with or without stabilizing protein component will mediate progressively stronger speck formation depending on the solubility of the calcium component. Thus, the addition of calcium and phosphate in established cell culture media will result in varying degrees of cellular calcium overload and biological effects depending on the overall composition, especially the protein content and thus the CPP forming propensity of cell culture media. Supplementary Figures 1, 2 corroborate this finding in that the effects of all calcification media were strictly dose-dependent over a range of 5 μg/ml calcium content (Supplementary Figure 1), 50 μg/ml (Supplementary Figure 2), and 100 μg/ml (Figure 7). Our systematic comparison of dose-dependent action of calcification media on real-time ASC inflammasome reporter macrophages underscores that seemingly minor changes in cell-based inflammation and calcification assays will result in different outcomes, which may help explain why individual published studies are almost impossible to compare.

**Figure 8:**
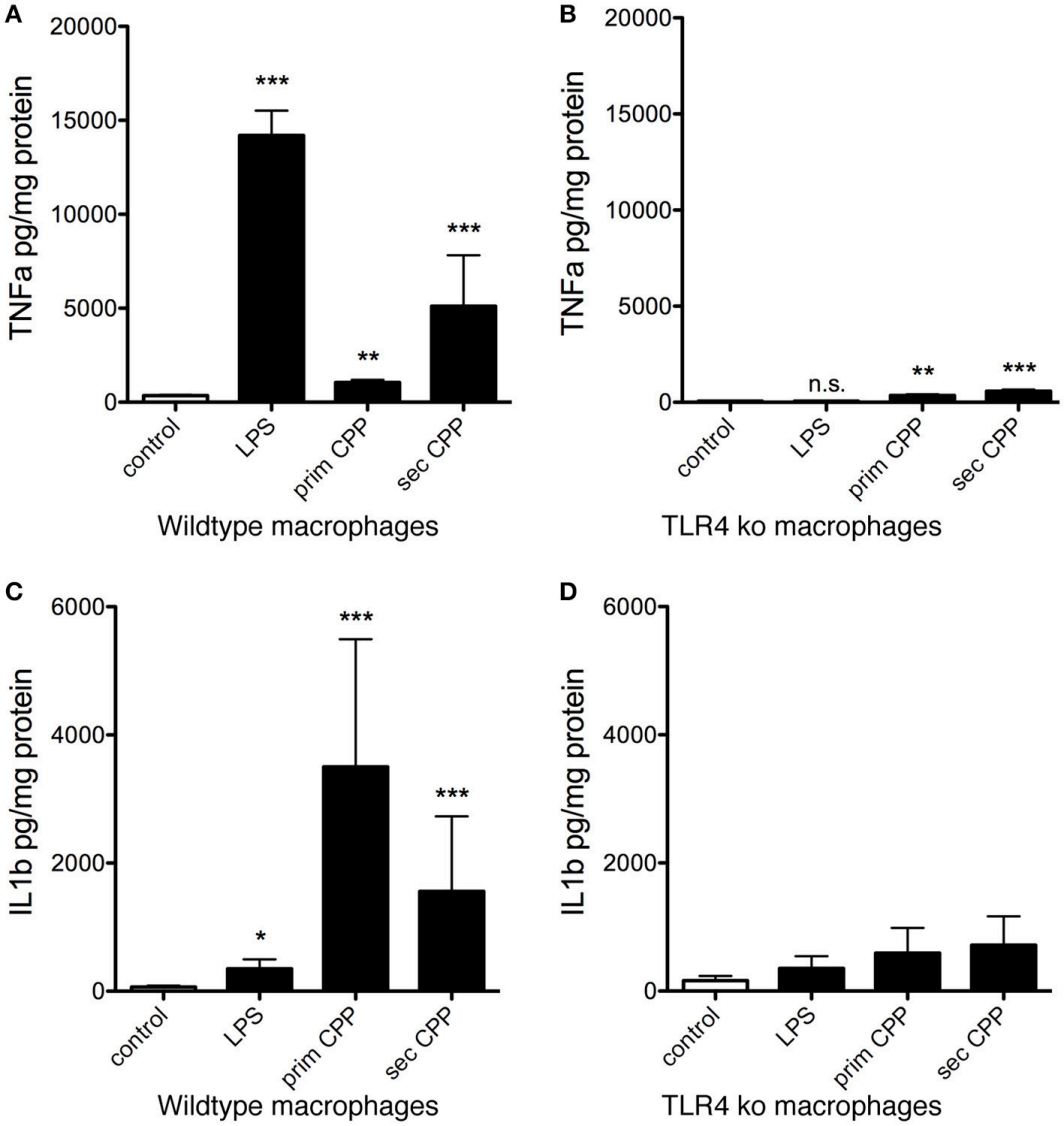
CPP-induced inflammatory cytokine secretion by macrophages is TLR4 dependent. Serum-starved wildtype and TLR4-deficient macrophages (TLR ko) where treated with LPS, primary or secondary CPP. **(A,B)** After 6 h stimulation, inflammatory cytokine TNFα secretion was determined in culture supernatants by ELISA. Secondary CPP caused stronger TNFα secretion than primary CPP. TLR4 ko macrophages showed 10-fold reduced TNFα secretion compared to wildtype macrophages suggesting a major contribution of TLR4 signaling in CPP-triggered TNFα secretion. Nevertheless, CPP-stimulated TLR4 ko still secreted higher amounts of TNFα compared to untreated control (prim CPP *p* < 0.01, sec CPP *p* < 0.001) suggesting a minor contribution to overall TNFα secretion of a TLR4-independent pathway. **(C,D)** After 16 h stimulation, supernatant IL-1β secreted by LPS-primed wildtype macrophages treated with primary CPP was twice as high as treated with secondary CPP. Both values were significantly higher than in wildtype macrophages treated with buffer control or with LPS. TLR4-deficient macrophages show a slight increase in IL-1β processing after the treatment with both types of particles. Overall, inflammatory cytokine secretion was strongly reduced in TLR4 ko. *p < 0.05, **p < 0.01, ***p < 0.001.

Next we measured inflammatory cytokine secretion following stimulation of wildtype and TLR4-deficient macrophages stimulated with LPS, primary or secondary CPP. Unlike the ASC-GFP reporter macrophages presented in Figure 7, Supplementary Figures 1, 2, these macrophages require priming for inflammasome activation and for IL-1β processing. Figures 8A,C show as expected LPS-triggered fast release of TNFα, but not of IL-1β a cytokine known to require a “second hit” for inflammasome activation, processing and secretion (32). Figures 8B,D show that TLR4-deficient macrophages secreted control levels of both TNFα and IL-1β confirming that TLR4 is the major signaling receptor for LPS. Primary CPP treatment of macrophages caused low level TNFα secretion, yet strong IL-1β secretion confirming that primary CPP triggered inflammasome activation (see Figure 8), which is critically required for IL-1β synthesis and secretion. In comparison to primary CPP, secondary CPP caused five-fold increased TNFα secretion indicating preferential stimulation of preformed cytokine secretion. On the other hand, primary CPP caused roughly two-fold higher IL-1β secretion compared to secondary CPP, indicating slower, yet longer lasting macrophage activation. Again these results mirror the inflammasome activation kinetics shown in Figure 7. We propose that the softer colloidal primary CPP due to their chemical instability, upon endocytosis and endosomal degradation cause cytoplasmic calcium increase, which triggers a robust and long-lasting inflammatory burst resulting in increased IL-1β secretion. In contrast the more rigid and crystalloid secondary CPP seem to predominantly trigger secretion of preformed TNFα.

## Discussion

With recent advances in analytical methods, the existence of calciprotein particles (CPP) that were long proposed on theoretical grounds and had been extensively studied *in vitro* have now been detected and characterized *in vivo*. In chronic kidney disease (CKD), CPP have emerged as potential nanoscale mediators of phosphate-induced toxicity (4, 12, 33). Similar to the situation in lipoprotein research several decades ago, ongoing research currently unravels CPP metabolism and the relevance of distinct forms of CPP in physiology and disease. We have previously demonstrated rapid blood clearance and contribution to cellular calcification of secondary CPP (14). We reasoned that *in vivo* however, the predominant form of CPP should be primary CPP. Recent work suggests that primary CPP and even earlier forms termed low density CPP—possibly as calciprotein monomers CPM (34)—are associated with CKD in patients who have a much increased risk of calcification (4). Here we demonstrate that primary and secondary CPP are predominantly cleared by endothelial cells and macrophages, respectively. Further the results of our study suggest that CPP-induced inflammation may be associated with cellular calcium overload triggering inflammasome activation and sustained inflammatory cytokine secretion. Thus, phosphate seems to be the driving force of CPP formation (1, 35–38), but calcium overload seems to be causing the inflammation-associated tissue damage and calcification associated with phosphate-induced damage. CPP mobilize excess calcium and phosphate as colloids also containing plasma-derived “mineral chaperone proteins” (5), thus indeed seem to be the “culprit of phosphorous woes” (38), especially if they are continuously formed, yet insufficiently cleared. This scenario is reminiscent of chronic dyslipidemia and the associated high levels of atherogenic lipoprotein complexes, which serve vital carrier function in normal physiology, but turn into disease promoting entities when abundant and modified by e.g., oxidation.

We demonstrated *in vivo* that both LSEC and Kupffer cells clear primary CPP within minutes from the sinusoidal circulation. LSEC play an important role in rapid clearance of primary CPP, in contrast to secondary CPP that are not rapidly taken up by these cells. This confirms the results of experiments with HUVEC and haEC that likewise preferentially cleared primary over secondary CPP corroborating a role of endothelial cells in the clearance of primary CPP. We speculated that CPP metabolism in healthy subjects is most likely limited to the bone remodeling compartment (6, 39), and to transport epithelia involved in bulk fluid and electrolyte transport like kidney, pancreas, lung, milk glands, placenta, and choroid plexus to name a few. Incidentally these tissues are prone to calcification, especially during inflammatory episodes. We propose that in CKD, the profound disturbance of mineral homeostasis will trigger formation of supraphysiological levels of circulating CPP (4, 25) driving clearing cells into a vicious cycle of CPP clearance and inflammation, a situation further compounded by hallmark pro-inflammatory stimuli associated with dialysis, namely uremic toxins and endotoxin. Thus, endothelial cells should be especially vulnerable to phosphate-associated toxicity in CKD; indeed endothelial dysfunction is possibly one of the earliest manifestations of vascular disease in CKD and other vasculopathies. Strong experimental evidence in fact vindicates phosphate-induced endothelial damage *in vitro* and *in vivo* (40–42). An interventional study reported reduced endothelial damage following dietary phosphate restriction (43), and the use of non-calcium-based phosphate binder use sevelamer has been associated with improvement in endothelial function (FMD) in hyperphosphataemic CKD stage 4 patients (44).

A major finding of this study was that only primary CPP are rapidly cleared by endothelial cells, while primary and secondary CPP are taken up by macrophages. We did not identify an endothelial cell receptor for primary CPP in this work, but positive identification of the clearance trajectory of early CPP forms may allow therapeutic intervention in CPP clearance. Differential clearance could be due to differences in physical properties of primary vs. secondary CPP, the latter being larger and more crystalline triggering phagocytosis on top of receptor mediated endocytosis. Macrophage recognition of crystals and nanoparticles is known to trigger NLPR3 inflammasome activation as described here (45). We hypothesize that distinct surface receptors will bind the respective protein corona of primary and secondary CPP, engaging differentially expressed clearance receptors in endothelial cells and macrophages. Recent proteomic analyses have revealed that secondary CPP, in particular, show a marked enrichment for the soluble apolipoproteins (e.g., ApoA1) and complement factors that may potentially serve as ligands for particle uptake (12). In addition, primary and secondary CPP may also be discriminated by their size and crystallinity. Both parameters are crucial in macrophage recognition of crystals and nanoparticles, as well as the respective cellular trajectories and signaling pathways engaged (45), and may even drive receptor-independent internalization.

We showed that primary CPP triggered inflammasome speck formation four-fold stronger than secondary CPP. Given their chemical instability and greater solubility under the conditions of endosomal degradation, we suggest that endolysosomal CPP degradation will cause cytoplasmic calcium release, thus triggering inflammasome activation. The relative contributions to NLRP3 activation of increased cytosolic [Ca2+] vs. decreased cytosolic [K+] remain disputed and incompletely defined (46). However, the dose-dependence of CPP action, and control experiments with matched extracellular calcium, which mimicked at high dose the strong NLRP3 inflammasome induction attained with microbial potassium ionophore nigericin, support the notion that CPP stability may determine their inflammatory potential. Cell-specific differential sensitivity may also apply. Bone researchers and tissue engineers routinely employ “osteogenic,” mineral-enriched cell culture media to coax mesenchymal precursor cells into an osteoblastic phenotype producing bone-like mineralized matrix. These “professional” mineralizing cells usually form mineralized matrix resembling dystrophic calcification more than mineralized bone, nevertheless without overt signs of inflammation. Calcification researchers on the other hand employ similar or even identical cell culture media to drive cells, e.g., smooth muscle cells, which do not normally mineralize, into pathological calcification. This latter process is usually associated with inflammation. It is presently unclear, what drives this inflammation. Our study suggests that excess mineral uptake and degradation of primary CPP by non-professional mineralizing cells may cause intracellular calcium-triggered inflammasome activation and thus inflammation. Side-by-side comparative studies of professional mineralizing osteoblastic cells and non-professional mineralizing cells including endothelial cells, epithelial cells and smooth muscle cells, which are all known to calcify in CKD, should clarify, if differential sensitivity to cellular calcium load indeed exists. Analyzing gene regulatory networks in these cells should tell if the differential sensitivity is reduced by osteochondrogenic conversion, which is a hallmark of e.g., calcifying smooth muscle cells. We strongly suggest to analyze, apart from cell viability and gene expression, inflammasome activation, a corollary of CPP clearance observed for the first time in this study. Several previous studies have evaluated the effect of calcium phosphate nanoparticles on inflammatory pathways and cell viability. However, relatively few have assessed the effects of biologically native protein-mineral complexes. Of these reports, both FCS and human serum-derived synthetic nanoparticles have been shown to stimulate IL-1β secretion from LPS-primed human monocytes and differentiated macrophage, depending on the length of exposure and on particle size (20, 23, 47). Calcium phosphate or apatite particles present a particularly adsorptive matrix, which may accrue and concentrate biologically active compounds from dilute solutions. Endotoxin even in low amounts is particularly notorious in confounding inflammatory cell assays with nanoparticles (47) and endotoxin contained in uremic plasma could certainly exacerbate the pro-inflammatory capacity of CPP. The plasma protein fetuin-A a major component of CPP, has been described *per se* as an endogenous TLR4 ligand triggering inflammation (48). A recent study on bone marrow lympho-myeloid malfunction in obesity questioned the role of fetuin-A as a physical adapter between TLR4 and dietary saturated fats (49) and earlier studies showed that LPS-free fetuin-A is anti-inflammatory (21) and protects against vascular smooth muscle calcification (50). Commercial fetuin-A preparations are however, impure and usually contain a range of biologically active compounds (51), which may include endotoxin. During this study we routinely tested for the presence of LPS in protein preparations using a high sensitivity endotoxin assay and discarded all preparations containing more than 0.1 EU/ml, a level considered safe for parenteral application in humans. Thus, we detected control level cytokine production and inflammasome activation when cells were treated with fetuin-A protein alone, with a dose identical to the one contained in the respective CPP preparations, effectively ruling out TLR4 binding and pro-inflammatory cell activation by fetuin-A protein under our experimental conditions. Nevertheless, it will be interesting to determine what is the ligand *in vivo* priming TLR4, which is necessary for full induction of the canonical inflammasome. Macrophages studied here were either intrinsically primed for inflammasome induction (Figure 7, Supplementary Figures 1, 2) or were LPS-primed for the study of IL-1β secretion. Virtually all particulate inflammasome agonists (silica, urate crystals, cholesterol crystals, alum etc.) only provide the activation signal and require priming by an additional independent stimulus—usually achieved with microbial TLR agonists like LPS *in vitro* (29, 52, 53). Oxidized LDL has been described as one of the few examples of a particle to provide both signals (54), but this was considered controversial due to the ever-present possibility of very low-level endotoxin contamination in reagents (55, 56). Indeed, we recently showed that CPP can bind and effectively concentrate endotoxin from uraemic serum (12) and thus at least in CKD, CPP may combine phosphate, calcium and endotoxin to effectively execute what is well-known as phosphate/inflammation associated endothelial damage ultimately resulting in dramatically increased CVD in CKD patients.

Dietary phosphate reduction and gut phosphate binding is best practice clinical routine in the treatment of CKD patients. Despite these measures CKD patients still have dramatically increased risk of inflammation and vascular calcification. Blood tests performed in several independent laboratories including our laboratories show that cardiovascular risk and calcification propensity are associated with CPP formation (4, 12, 25, 27, 57–61). Clinical CPP monitoring is not routinely done, because blood testing for calcification propensity is currently not universally available. Monitoring and reducing CPP levels and regulating their cellular uptake in the dialysis-free interval may however, further improve outcome in dialysis patients in addition to phosphate control.

## Author contributions

SK, ABü and ES performed experiments, analyzed data, drafted and revised the manuscript. ABa, EL, and JH designed the study, and revised the manuscript. AG performed experiments, analysed data, and revised the manuscript. EB performed experiments. WJ-D designed the study, analyzed data, drafted, and revised the manuscript.

### Conflict of interest statement

The authors declare that the research was conducted in the absence of any commercial or financial relationships that could be construed as a potential conflict of interest.

## Supplementary material

Supplementary Table 1 Chemical and pharmacological inhibitors used to study CPP endocytosis.

Supplementary Movie 1 Functional imaging of primary CPP clearance using 2-photon intravital microscopy.

Supplementary Movie 2 Functional imaging of secondary CPP clearance using 2-photon intravital microscopy.

Supplementary Figure 1 Calcification media induce differential cellular calcium overload and inflammasome activation. Immortalized macrophages expressing the inflammasome adaptor protein labeled *apoptosis-associated speck like protein containing a caspase recruitment domain* ASC fused to *green fluorescent protein*, were exposed to buffer control, LPS, nigericin, or to calcification media with elevated calcium or phosphate, individually and in combination, as well as with primary or secondary CPP. Notably, all calcification media contained an identical total calcium dose of 5 μg, yet differed in the stability of the calcium containing mineral component. Scale bar 25 μm.

Supplementary Figure 2 Calcification media induce differential cellular calcium overload and inflammasome activation. Immortalized macrophages expressing the inflammasome adaptor protein labeled *apoptosis-associated speck like protein containing a caspase recruitment domain* ASC fused to *green fluorescent protein*, were exposed to buffer control, LPS, nigericin, or to calcification media with elevated calcium or phosphate, individually and in combination, as well as with primary or secondary CPP. Notably, all calcification media contained an identical total calcium dose of 50 μg, yet differed in the stability of the calcium containing mineral component. Scale bar 25 μm.

Supplementary Figure 3 Differential clearance of primary and secondary CPP. Mice were injected with fluorescence labeled fetuin-A monomer (red), primary CPP (red), secondary CPP (green), or a mixture of primary (red), and secondary CPP (green) and liver, spleen, pancreas, kidney, lung, heart, and brown adipose fat tissue (BAT) were harvested 10 min after injection, sectioned, and analyzed for the presence of CPP by fluorescence microscopy. Liver and spleen stained positive for primary and secondary CPP, while all other organs stained negative. All organs stained negative for fetuin-A monomer. Considering the relative tissue mass of liver vs. spleen, therefore the liver can be considered the major clearance organ for CPP.

Supplementary Figure 4 Differential endocytosis of fetuin-A monomer, primary and secondary CPP by bone marrow-derived macrophages. To discriminate fetuin-A and CPP binding from endocytosis we incubated bone marrow-derived macrophages with fetuin-A monomer, primary and secondary CPP at 4°C to assess binding, or at 37°C to assess endocytosis. Following 1 h of incubation we analyzed cell-associated fluorescence by flow cytometry. Incubation at 4°C yielded much lower cell-associated fluorescence than incubation at 37°C, especially for primary and secondary CPP. These results indicate that the cells readily endocytosed secondary CPP, and less well also primary CPP. A small amount of fetuin-A monomer was also endocytosed.
