# Supplementary material for: Cellular Clearance and Biological Activity of Calciprotein Particles Depend on Their Maturation State and Crystallinity
Source: Front Immunol. 2018 Sep 4;9:1991. doi: 10.3389/fimmu.2018.01991 (PMC6131296; doi:10.3389/fimmu.2018.01991)
Supplement: Supplementary Table 1 — Chemical and pharmacological inhibitors used to study CPP endocytosis. [file Table_1.DOCX]

**Supplementary Table 1.** Chemical and pharmacological inhibitors used to study CPP endocytosis.

| Inhibitor | Action | Final [Conc.] | Ref. |
| --- | --- | --- | --- |
| cytochalasin D (cytoD) | blocks actin polymerisation; non-specific endocytic inhibitor | 10 μM | (Ivanov, 2008) |
| chlorpromazine  (CPMZ) | inhibits clathrin-mediated endocytosis; may also block phagocytosis | 10 μg/ml | (dos Santos et al., 2011) |
| monodansylcadaverine (MDC) | inhibits clathrin-mediated endocytosis | 100 μM | (Wang et al., 2012) |
| filipin | inhibits lipid raft/caveolae-mediated endocytosis | 1 μM | (Ros-Baro et al., 2001) |
| genistein | inhibits caveolae-mediated endocytosis | 200 μM | (dos Santos et al., 2011) |
| methyl-β-cyclodextrin (MβCD) | inhibits lipid raft/caveolae-mediated endocytosis; may also block clathrin-and fluid phase-mediated endocytosis | 4 mM | (Vercauteren et al., 2010) |
| dimethyl amiloride (DMA) | blocks constitutive and stimulated macropinocytosis/phagocytosis | 50 μM | (Nakase et al., 2004) |
| LY290042 | blocks phosphatidylinositol-3-kinase-dependent constitutive and stimulated macropinocytosis/phagocytosis | 20 μM | (Montaner et al., 1999) |
| polyinosinic acid (polyI) | inhibits class A scavenger receptor | 10 μg/ml | (Thelen et al., 2010) |
