# Supplementary figures and images for: Cellular Clearance and Biological Activity of Calciprotein Particles Depend on Their Maturation State and Crystallinity
Source: Front Immunol. 2018 Sep 4;9:1991. doi: 10.3389/fimmu.2018.01991 (PMC6131296; doi:10.3389/fimmu.2018.01991)

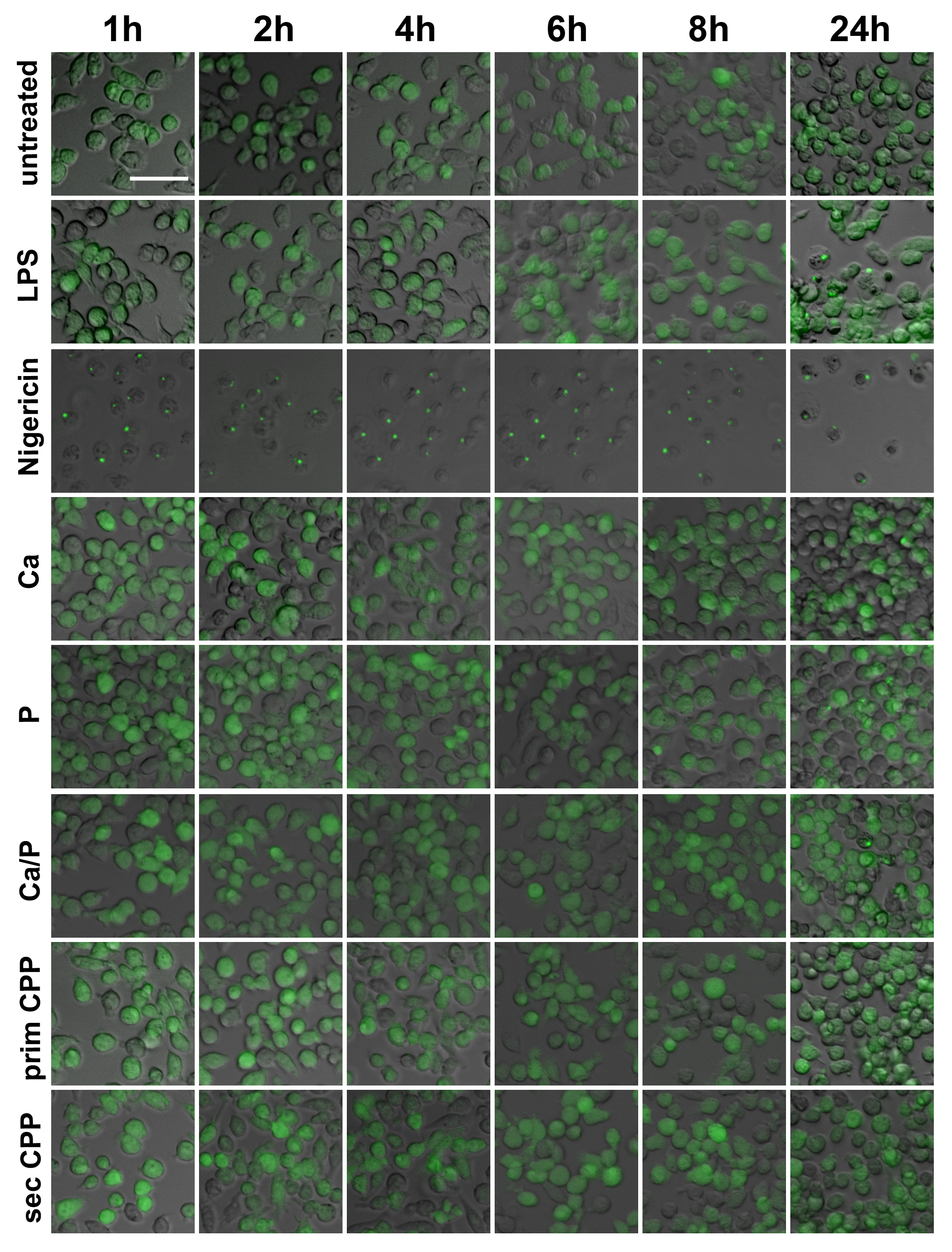

Supplement: Supplementary Figure 1 — Calcification media induce differential cellular calcium overload and inflammasome activation. Immortalized macrophages expressing the inflammasome adaptor protein labeled apoptosis-associated speck like protein containing a caspase recruitment domain ASC fused to green fluorescent protein, were exposed to buffer control, LPS, nigericin, or to calcification media with elevated calcium or phosphate, individually and in combination, as well as with primary or secondary CPP. Notably, all calcification media contained an identical total calcium dose of 5 μg, yet differed in the stability of the calcium containing mineral component. Scale bar 25 μm. [file Image_1.TIF]

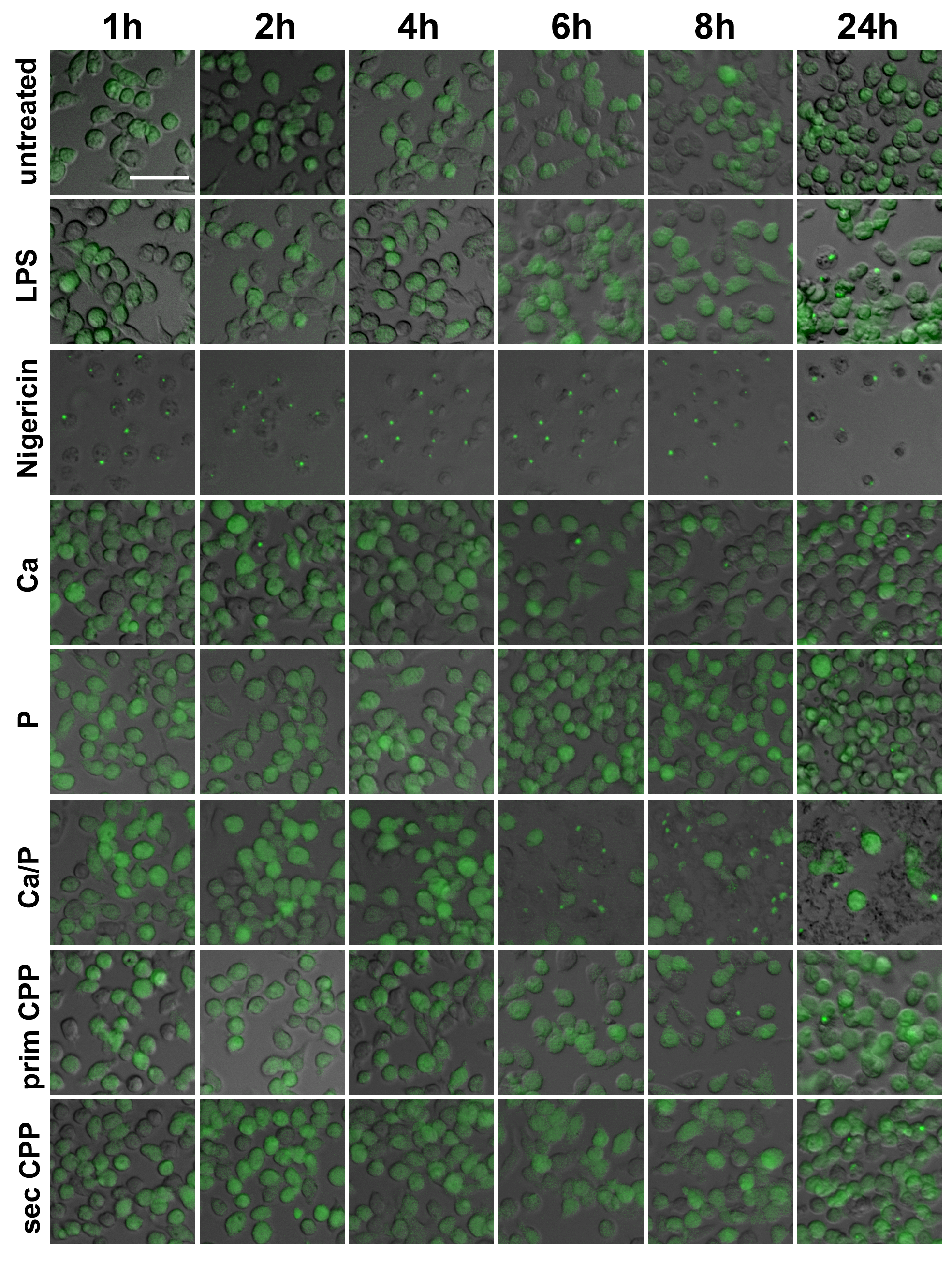

Supplement: Supplementary Figure 2 — Calcification media induce differential cellular calcium overload and inflammasome activation. Immortalized macrophages expressing the inflammasome adaptor protein labeled apoptosis-associated speck like protein containing a caspase recruitment domain ASC fused to green fluorescent protein, were exposed to buffer control, LPS, nigericin, or to calcification media with elevated calcium or phosphate, individually and in combination, as well as with primary or secondary CPP. Notably, all calcification media contained an identical total calcium dose of 50 μg, yet differed in the stability of the calcium containing mineral component. Scale bar 25 μm. [file Image_2.TIF]

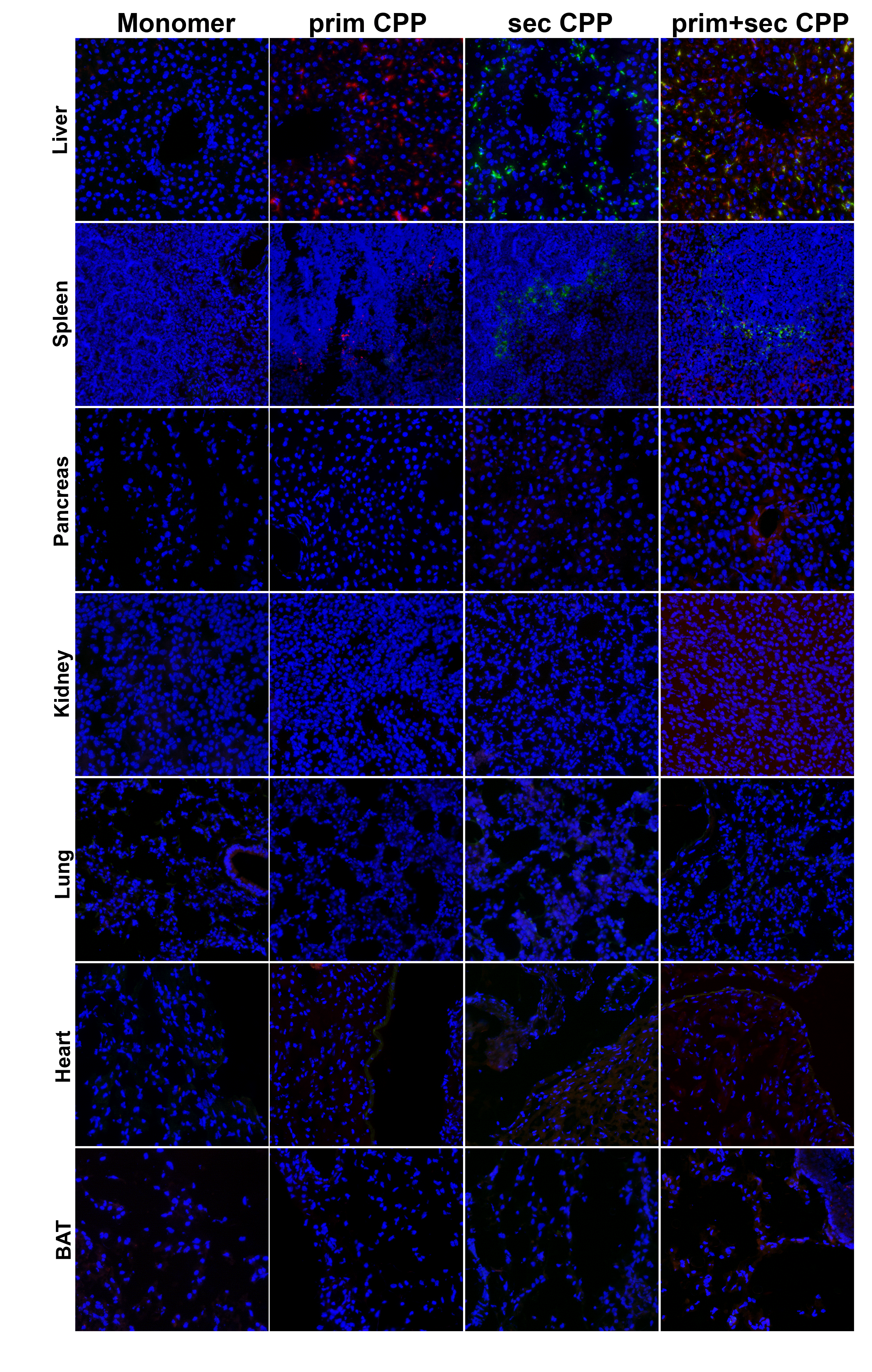

Supplement: Supplementary Figure 3 — Differential clearance of primary and secondary CPP. Mice were injected with fluorescence labeled fetuin-A monomer (red), primary CPP (red), secondary CPP (green), or a mixture of primary (red), and secondary CPP (green) and liver, spleen, pancreas, kidney, lung, heart, and brown adipose fat tissue (BAT) were harvested 10 min after injection, sectioned, and analyzed for the presence of CPP by fluorescence microscopy. Liver and spleen stained positive for primary and secondary CPP, while all other organs stained negative. All organs stained negative for fetuin-A monomer. Considering the relative tissue mass of liver vs. spleen, therefore the liver can be considered the major clearance organ for CPP. [file Image_3.PNG]

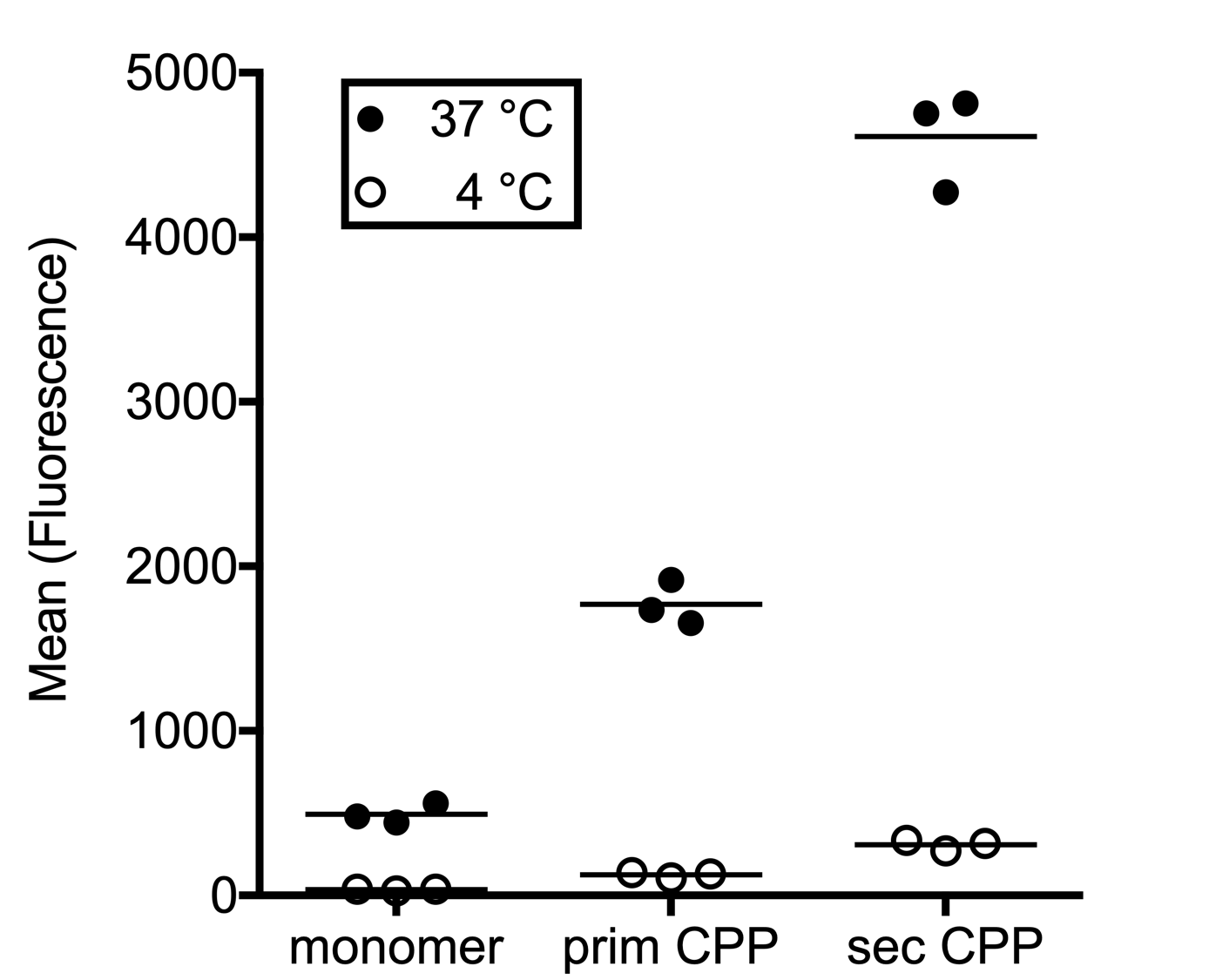

Supplement: Supplementary Figure 4 — Differential endocytosis of fetuin-A monomer, primary and secondary CPP by bone marrow-derived macrophages. To discriminate fetuin-A and CPP binding from endocytosis we incubated bone marrow-derived macrophages with fetuin-A monomer, primary and secondary CPP at 4°C to assess binding, or at 37°C to assess endocytosis. Following 1 h of incubation we analyzed cell-associated fluorescence by flow cytometry. Incubation at 4°C yielded much lower cell-associated fluorescence than incubation at 37°C, especially for primary and secondary CPP. These results indicate that the cells readily endocytosed secondary CPP, and less well also primary CPP. A small amount of fetuin-A monomer was also endocytosed. [file Image_4.PNG]
